# Ischemic Stroke and Traumatic Brain Injury: Epidemiology, Molecular Pathways, and Emerging Therapies

**DOI:** 10.1002/mco2.71053

**Published:** 2026-10-09

**Authors:** Mengzhao Feng, Qi Qin, Fang Wang, Kaiyuan Zhang, Zhihua Li, Junlei Chang, Min Yu, Fuyou Guo

**Affiliations:** ^1^ Department of Neurosurgery The First Affiliated Hospital of Zhengzhou University Zhengzhou City Henan Province China; ^2^ State Key Laboratory of Biomedical Imaging Science and System Institute of Biomedicine and Biotechnology Shenzhen Institute of Advanced Technology Chinese Academy of Sciences Shenzhen Guangdong Province China; ^3^ Department of Anatomy Basic Medical Sciences College Zhengzhou University Zhengzhou City Henan Province China; ^4^ Stroke Center Department of Neurology The First Hospital of Jilin University Changchun Jilin Province China; ^5^ Neuroscience Research Center The First Hospital of Jilin University Changchun Jilin Province China

**Keywords:** blood–brain barrier, clinical trials, ischemic stroke, neuroinflammation, programmed cell death, secondary brain injury, traumatic brain injury

## Abstract

Ischemic stroke and traumatic brain injury are two leading causes of death and long‐term disability worldwide. Despite their different primary insults, both conditions trigger a complex cascade of secondary brain injury that largely determines neurological outcome. This review systematically compares the epidemiology, shared molecular pathways, and emerging therapeutic strategies for secondary brain injury after ischemic stroke and traumatic brain injury. We focus on three interconnected pathological cascades spanning blood–brain barrier disruption, neuroinflammation, and neuronal death driven by excitotoxicity, oxidative stress, and multiple forms of programmed cell death. For each pathway, we highlight convergent and divergent mechanisms between the two disorders. We then summarize emerging preclinical interventions ranging from small molecule inhibitors and repurposed drugs to nanomedicine, extracellular vesicles, and neuromodulation techniques. In parallel, we review recent and ongoing clinical trials targeting these pathways, emphasizing mechanism‐based strategies. Finally, we discuss current challenges and offer future perspectives on biomarker‐driven patient stratification, innovative drug delivery systems bypassing the blood–brain barrier, and multitarget combination regimens. By providing a unified framework that compares ischemic stroke and traumatic brain injury side by side, this review aims to guide the development of more effective, personalized treatments for secondary brain injury.

## Graphical abstract

Schematic overview of secondary brain injury after ischemic stroke and traumatic brain injury. Both conditions trigger endothelial cell dysfunction, pericyte contraction, and upregulated AQP4 expression in astrocytic end‐feet, leading to blood–brain barrier disruption. This compromised barrier facilitates peripheral immune cell infiltration into the brain parenchyma. Concurrently, resident microglia polarize toward the proinflammatory M1 phenotype and astrocytes shift to the neurotoxic A1 phenotype, releasing inflammatory cytokines and chemokines that amplify neuroinflammation. These events further promote multiple forms of programmed neuronal death, including apoptosis, necroptosis, pyroptosis, and ferroptosis, together with excitotoxicity and oxidative stress. The interconnected cascades ultimately exacerbate secondary brain injury and neurological deterioration.

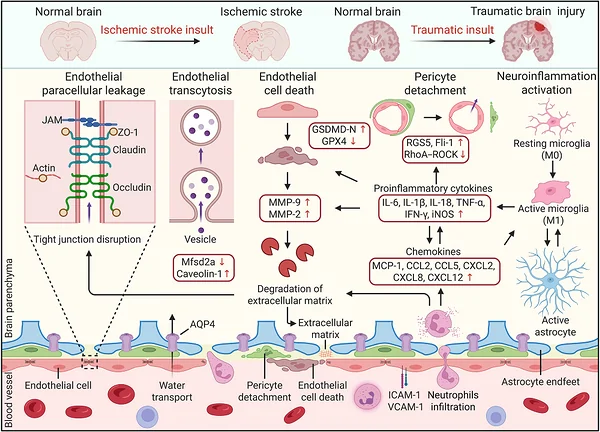

## 1 Introduction

Ischemic stroke (IS) and traumatic brain injury (TBI) are the most devastating neurological disorders worldwide, together affecting more than 60 million people each year and imposing an enormous socioeconomic burden [1, 2, 3]. IS, typically caused by thromboembolic occlusion of a cerebral artery, is the second leading cause of death and a primary driver of long‐term disability [4, 5]. TBI, in contrast, results from an external mechanical force to the head, with an estimated 50–60 million new cases annually, particularly in low‐ and middle‐income countries (LMICs) due to road traffic incidents [6, 7]. Despite these fundamentally different origins, the two conditions share a critical pathological feature in that the primary insult sets off a complex cascade of secondary brain injury that largely dictates neurological deterioration and clinical outcome.

The secondary injury cascades triggered by IS and TBI are extraordinarily complex. They involve a dynamic interplay of blood–brain barrier (BBB) disruption, neuroinflammation, oxidative stress, excitotoxicity, and multiple forms of programmed cell death [8, 9, 10, 11, 12, 13, 14, 15, 16]. These processes do not occur in a linear or temporally fixed sequence. Instead, they evolve with distinct spatial and temporal profiles that vary considerably among individuals [17, 18]. Furthermore, extensive cross‐talk among these pathways creates positive feedback loops that amplify tissue damage and extend beyond the initial lesion [19, 20]. As a result, the pathological evolution is highly heterogeneous. The clinical course is often unpredictable, with outcomes ranging from full recovery to severe disability or death depending on factors such as lesion location, severity, and timely access to reperfusion therapies in the case of IS. While revascularization strategies have markedly improved outcomes in selected IS patients, TBI still lacks specific mechanism‐based pharmacological interventions, and many patients suffer progressive neurological deterioration and long‐term disability despite optimal supportive care [21, 22]. These challenges underscore the urgent need for a deeper, integrated understanding of their shared and divergent pathophysiology.

In this review, we first present the global epidemiology and disease burden of IS and TBI, highlighting their increasing impact on healthcare systems and the urgent need for improved prevention and management. We then systematically dissect the core molecular pathways of secondary brain injury common to both disorders, including BBB disruption, neuroinflammation, and neuronal death. For each pathway, we compare the underlying mechanisms between IS and TBI, emphasizing both convergences and divergences. Subsequently, we review emerging therapeutic strategies that have shown preclinical or clinical efficacy, organized by the same pathophysiological categories to facilitate cross‐condition comparison. Finally, we address current challenges, including the narrow therapeutic time window, disease heterogeneity, and limitations of single‐target interventions. We offer future perspectives on advanced drug delivery systems, biomarkers for patient stratification, and the potential of multitargeted, personalized therapies. By systematically comparing IS and TBI, this review aims to provide a novel framework for developing more effective treatments that leverage their convergent pathophysiology.

## 2 Epidemiology and Disease Burden

### 2.1 IS Epidemiology and Disease Burden

According to the Global Burden of Disease (GBD) 2021 study, IS accounts for approximately 7.8 million new cases worldwide each year, representing about 65% of all strokes, with a global age‐standardized incidence of 92.4 per 100,000 population [23, 24]. The prevalence has steadily risen to an estimated 69.9 million cases globally due to population aging and improved poststroke survival [25]. Globally, stroke accounted for 7.3 million deaths and 160.5 million disability‐adjusted life years (DALYs), of which IS contributed 3.6 million deaths and 70.4 million DALYs. High systolic blood pressure accounted for approximately 58% of the DALYs attributable to IS [26], establishing it as the single most important preventable cause of both stroke mortality and long‐term disability. The burden extends well beyond acute mortality, driving long‐term disability, high healthcare costs, and caregiver dependence. Although age‐standardized mortality has declined in high‐income countries (HICs), it has stagnated or risen in many LMICs where access to revascularization therapies and secondary prevention remains limited [27]. This unevenly distributed burden underscores the urgent need for effective prevention, equitable acute care, and long‐term rehabilitation worldwide.

### 2.2 TBI Epidemiology and Disease Burden

According to the GBD 2021 study, TBI is a leading cause of death and disability worldwide, with approximately 20.8 million new cases and 37.9 million prevalent cases occurring annually [28]. The global age‐standardized incidence of TBI was estimated to be 259 per 100,000 population, with substantial variation by region and income level [29]. The epidemiological profile of TBI is shifting. In HICs, falls have surpassed road traffic incidents as the leading cause, especially among adults aged 65 years or older [30, 31]. In LMICs, road traffic collisions remain the predominant mechanism [32, 33]. Globally, TBI contributes to approximately 5.48 million years lived with disability and a similar magnitude of years of life lost, with mortality rates highest in LMICs where access to acute care is limited [28].

TBI is increasingly recognized as a chronic disease [34]. Notably, more than 90% of hospital‐presenting TBI cases are mild, defined as Glasgow Coma Scale (GCS) scores of 13–15, yet nearly half of these patients fail to achieve full recovery by 6 months [6, 35]. Many survivors suffer persistent cognitive, behavioral, and mood disorders [36, 37, 38, 39, 40, 41]. Moderate (GCS 9–12) and severe (GCS 3–8) TBI cases, though less frequent, contribute disproportionately to mortality and long‐term disability, with only 16% of patients with moderate‐to‐severe TBI achieving complete functional recovery at 6 months postinjury [42, 43, 44]. TBI also increases the risk of late‐onset neurodegenerative diseases including Alzheimer's disease, Parkinson's disease, and chronic traumatic encephalopathy [45, 46, 47, 48]. Beyond these well‐recognized associations, accumulating evidence links TBI to the accumulation of hyperphosphorylated tau and TDP‐43 pathology, as well as to progressive white‐matter degeneration, which may contribute to delayed cognitive decline and motor impairment in survivors [49, 50, 51, 52, 53, 54, 55, 56]. Together, these findings underscore the urgent need for improved prevention, standardized acute management, and accessible postacute rehabilitation, particularly in LMICs and for vulnerable populations especially the elderly.

## 3 Shared Core Molecular Pathways of Secondary Brain Injury

In both IS and TBI, the primary insult triggers a cascade of secondary injury mechanisms that are highly interconnected and self‐amplifying. This section systematically dissects three core pathological pillars comprising BBB disruption, neuroinflammation, and neuronal death, which represent the major drivers of neurological deterioration. We first describe how BBB breakdown serves as the initial gateway, allowing peripheral immune infiltration and parenchymal exposure to neurotoxic factors. Next, we discuss the ensuing neuroinflammatory response, driven by activated microglia, reactive astrocytes, and infiltrating immune cells, which perpetuates a hostile microenvironment. Finally, we examine the terminal pathways of neuronal injury, including multiple forms of programmed cell death, excitotoxicity, and oxidative stress. For each process, we compare convergent and divergent mechanisms between IS and TBI, providing a unified framework for understanding secondary brain injury.

### 3.1 BBB Disruption

The BBB is a highly specialized structural and functional interface that maintains central nervous system homeostasis by strictly regulating the exchange of molecules between the blood and the brain parenchyma [15, 57, 58]. The BBB is composed of brain microvascular endothelial cells, which are connected by tight junction (TJ) complexes, along with pericytes embedded in the basement membrane and astrocytic end‐feet that collectively form the neurovascular unit [59, 60, 61, 62]. Under physiological conditions, the BBB effectively prevents the entry of neurotoxic plasma components, blood cells, and pathogens into the brain. However, in both IS and TBI, the primary insult triggers a cascade of events that rapidly and progressively dismantle the BBB [38, 63, 64].

#### 3.1.1 Endothelial Cell Dysfunction

Endothelial dysfunction represents the central event in BBB disruption following both IS and TBI. However, the primary triggers differ between the two conditions. In IS, energy failure due to oxygen‐glucose deprivation directly compromises endothelial ion homeostasis, leading to cellular edema, acidification, and the generation of reactive oxygen species (ROS) from mitochondria and xanthine oxidase. These early metabolic disturbances initiate a cascade of endothelial injury even before reperfusion occurs [65]. Upon reperfusion, a surge in oxidative burst and rapid inflammatory signaling further amplifies endothelial damage [66]. In TBI, mechanical impact causes immediate physical disruption of the microvascular endothelium, including membrane tearing, shear stress‐induced deformation, and direct endothelial cell loss. This is rapidly followed by extravasation of blood components, including hemoglobin and thrombin, which trigger inflammatory and oxidative cascades. Concurrently, the release of damage‐associated molecular patterns (DAMPs) activates surrounding glia and endothelial pattern recognition receptors, initiating a sterile inflammatory response that sustains barrier injury [67, 68]. Despite these distinct initiating events, both conditions converge on three interrelated mechanisms of endothelial dysfunction, including paracellular hyperpermeability due to TJ degradation, enhanced caveolae‐mediated transcytosis, and programmed cell death of brain microvascular endothelial cells. Each of these mechanisms is discussed in detail below.

Following IS and TBI, brain microvascular endothelial cells undergo profound dysfunction, leading to BBB breakdown through paracellular hyperpermeability, increased transcytosis, and cell death [69, 70]. Paracellular disruption, driven by the degradation of TJ proteins, is a central mechanism. In both IS and TBI, the TJ proteins Claudin‐5, occludin, and zonula occludens‐1 (ZO‐1) are rapidly downregulated or redistributed, directly compromising the paracellular seal [71, 72]. This process is largely mediated by matrix metalloproteinases (MMPs), particularly MMP‐9 [73]. In IS, ischemia–reperfusion triggers a surge in MMP‐9 activity, which proteolytically cleaves TJ proteins and the extracellular matrix [74]. Similarly, in TBI, mechanical injury and subsequent neuroinflammation upregulate MMP‐9 via the IL‐17 signaling pathway, leading to TJ degradation and increased vascular permeability [75].

Beyond MMP‐mediated TJ degradation, emerging evidence has identified the sphingosine‐1‐phosphate (S1P)/S1P receptor (S1PR) signaling axis as a major regulator of endothelial stability and vascular permeability in both IS and TBI [76]. S1P, a bioactive sphingolipid metabolite, signals through five G protein‐coupled receptors (S1PR1–S1PR5) to regulate diverse cellular functions, including endothelial barrier integrity, lymphocyte trafficking, and inflammatory responses [76]. Among these, S1PR1 and S1PR2 play opposing roles in modulating BBB function. Endothelial S1PR1 activation preserves barrier integrity by promoting adherens junction assembly and limiting caveolae‐mediated vesicular transcytosis [77]. In TBI models, selective S1PR1 agonism significantly attenuated BBB disruption by restoring major facilitator superfamily domain‐containing protein 2a (Mfsd2a) expression and reducing Caveolin‐1 (Cav‐1)‐mediated vesicular transport, thereby preserving TJ integrity and improving neurological outcomes [77]. Conversely, S1PR2 activation disrupts endothelial barrier function through Rho/ROCK‐dependent cytoskeletal contraction and caveolin‐mediated endocytosis of VE‐cadherin, leading to adherens junction disassembly and loss of endothelial polarity [78]. Notably, S1PR2 expression exhibits sexually dimorphic patterns in CNS autoimmunity and correlates with increased BBB permeability in disease‐susceptible regions [78].

Recent sphingolipidomic profiling has further elucidated the temporal dynamics of S1P and related lipid species in the peri‐infarct tissue after IS [79]. Within 6 h of ischemia onset, S1P levels are significantly reduced while ceramide species, including C16, C18, and C24:1 ceramides, are markedly elevated [79]. These early lipid alterations correlate with endothelial destabilization and an increased risk of hemorrhagic transformation. Moreover, specific sphingolipid species, particularly C18 lactosylceramide and C24:1 ceramide, are associated with enhanced immune cell infiltration and activation of CNS‐resident cells, providing a mechanistic link between BBB breakdown and downstream neuroinflammatory cascades [79]. Upstream signaling pathways, including the RhoA/ROCK, PKC, and MAPK cascades, are activated in IS and TBI, leading to TJ protein phosphorylation, internalization, and cytoskeletal rearrangement [80, 81, 82, 83]. Concurrently, proinflammatory cytokines such as TNF‐α and IL‐1β, released from activated glia and infiltrating immune cells, further suppress the expression of Claudin‐5 and occludin [84, 85]. While transcellular caveolar transport also increases, the disruption of TJ represents a critical and convergent molecular hub for BBB failure in both IS and TBI.

Transcytosis that is enhanced via caveolae plays an early and dominant role in breaking down the BBB in both IS and TBI [86, 87]. This process often precedes TJ disruption. Under normal conditions, brain endothelial cells express Mfsd2a. Mfsd2a transports lysophosphatidylcholine and creates a unique lipid environment that suppresses caveolae formation [88]. After IS or TBI, Mfsd2a expression is significantly downregulated. This downregulation relieves the inhibition of caveolae biogenesis and leads to a rapid increase in vesicular transcytosis [89, 90]. Concurrently, Cav‐1 expression is upregulated [91, 92]. Src family kinases then phosphorylate Cav‐1 at tyrosine residue‐14. This phosphorylation event promotes caveolae budding and intracellular trafficking [93].

In IS, the biphasic increase in BBB permeability begins with a surge in caveolae‐mediated transcytosis, whereas TJ proteins remain ultrastructurally intact [94]. Endothelial glycocalyx degradation further accelerates this process. Glycocalyx breakdown enhances the interaction between Syndecan‐1 and Src through the Src SH2 domain, leading to Src‐dependent Cav‐1 phosphorylation and subsequent caveolae internalization [93]. In TBI, mechanical injury similarly reduces Mfsd2a expression and elevates Cav‐1 levels [90, 95]. This results in abundant albumin‐filled vesicular trafficking across brain endothelial cells [77]. Moreover, Mfsd2a deficiency impairs S1P export from endothelial cells. The resulting decrease in perivascular S1P concentration reduces S1PR1 signaling. Diminished S1PR1 activity further disinhibits vesicle formation and exacerbates transcellular permeability [77]. Collectively, the Mfsd2a/Cav‐1 axis constitutes a convergent molecular hub that drives pathological transcytosis and early BBB disruption in both IS and TBI.

Programmed death of brain microvascular endothelial cells constitutes a direct and critical mechanism of BBB breakdown in both IS and TBI [96, 97]. After IS, oxygen‐glucose deprivation triggers mitochondrial pathway apoptosis in brain microvascular endothelial cells, characterized by cytochrome *c* release, apoptosome formation, and Caspase‐3 activation [98, 99]. TNF‐α derived from activated microglia engages the extrinsic pathway and drives necroptosis via RIPK1–RIPK3–MLKL signaling, leading to membrane permeabilization and barrier loss [96, 100]. In the hyperglycemic ischemic milieu, the long noncoding RNA Meg3 suppresses glutathione peroxidase 4 (GPX4) transcription through p53, inducing endothelial ferroptosis with iron‐dependent lipid peroxidation and TJ degradation [101]. Pyroptosis is also engaged, as hypoxia‐induced ROS activate NLRP3 inflammasomes, causing Caspase‐1‐mediated GSDMD cleavage, pore formation, and release of IL‐1β/IL‐18, which amplifies endothelial injury and BBB hyperpermeability [82, 102].

In TBI, mechanical impact directly damages brain microvascular endothelial cells and triggers secondary cascades. Ferroptosis is prominent, with GPX4 downregulation, Cyclooxygenase‐2 upregulation, and iron accumulation, all reversed by the ferroptosis inhibitor Ferrostatin‐1, which restores TJ proteins ZO‐1 and occludin [103]. Neutrophil extracellular traps (NETs) activate the TLR4/NF‐κB pathway in brain endothelial cells, inducing GSDMD‐dependent pyroptosis and subsequent BBB disruption [104]. In addition, elevated CXCL‐10 expression in brain microvascular endothelial cells activates Caspase‐1, leading to GSDMD cleavage and pore formation. This pyroptotic cascade promotes the release of IL‐1β and IL‐18, amplifies endothelial injury, and directly compromises BBB integrity [105]. Blast injury upregulates Complex I genes and P2RX7 in brain microvascular endothelial cells, causing mitochondrial Ca^2^^+^ overload and ROS production. This compromises mitochondrial membrane integrity, activates Caspases‐1, ‐7, ‐9 and downstream Caspase‐3/7, ultimately triggering endothelial apoptosis and BBB disruption [106, 107]. Endothelial‐specific deletion of IRE1α sensitizes brain microvascular endothelial cells to injury‐induced death, as evidenced by increased immune infiltration and neuronal apoptosis, indicating that IRE1α protects against endothelial loss after TBI [64]. While apoptosis, pyroptosis, and ferroptosis are shared mechanisms of endothelial injury in both IS and TBI, necroptosis has been reported in IS but not consistently observed in TBI endothelial cells. Nevertheless, all these pathways converge to dismantle endothelial integrity and exacerbate neurological outcomes.

#### 3.1.2 Pericyte Dysfunction

Pericytes are indispensable for BBB integrity, and their dysfunction or death critically contributes to BBB breakdown in both IS and TBI [71, 92, 108]. In IS, pericyte detachment from microvessels is driven by hypoxia‐inducible factor‐1α (HIF‐1α)‐mediated upregulation of regulator of G‐protein signaling 5 (RGS5), which reduces vascular coverage and disrupts TJ proteins [109, 110, 111]. Concurrently, pericyte ferroptosis occurs after hemorrhagic conversion, characterized by iron overload, GPX4 inactivation, and lipid peroxidation, directly compromising BBB integrity [112]. In TBI, mechanical injury upregulates the transcription factor Fli‐1 in pericytes. Fli‐1 upregulation is closely associated with pericyte apoptosis and pericyte loss, which in turn compromise BBB integrity [113]. Moreover, TBI suppresses the integrin α8 (ITGA8)–RhoA–ROCK axis in pericytes, impairing cytoskeletal organization and pericyte process extension, which in turn reduces activation of latent TGF‐β1 and downregulates TJ proteins including Claudin‐5 and occludin [114]. Accumulation of pathological proteins such as amyloid‐β (Aβ) and α‐synuclein further aggravates pericyte dysfunction. Specifically, Aβ induces Endothelin‐1‐mediated pericyte contraction and ROS‐dependent degeneration, while α‐synuclein triggers pericyte release of IL‐1β, IL‐6, TNF‐α, and MMP‐9, all of which disrupt BBB integrity [115]. Collectively, RGS5‐mediated detachment, Fli‐1‐driven cell death, ITGA8–RhoA–ROCK cytoskeletal failure, ferroptosis, and cytokine‐mediated inflammation converge to compromise pericyte survival and function, thereby exacerbating BBB disruption in IS and TBI.

#### 3.1.3 Astrocyte End‐Feet Dysfunction

Dysfunction of astrocytic end‐feet critically compromises BBB integrity [70, 116]. Under physiological conditions, astrocyte end‐feet completely ensheathe cerebral microvessels and maintain a polarized distribution of aquaporin‐4 (AQP4), which facilitates water and solute clearance via the glymphatic system [117, 118]. After IS or TBI, mechanical or hypoxic–ischemic injury triggers reactive astrogliosis, characterized by upregulation of glial fibrillary acidic protein (GFAP) and a marked increase in AQP4 expression [119]. However, this increased AQP4 is mislocalized, thereby losing its perivascular polarization and becoming diffusely distributed along the astrocyte membrane. The loss of AQP4 polarization is driven by downregulation of anchoring proteins such as α‐syntrophin and dystroglycan, which normally tether AQP4 to the end‐foot membrane facing the vessel wall [120].

Concurrently, injured neurons and reactive glia release high levels of vascular endothelial growth factor (VEGF). VEGF binds to VEGF receptor‐2 (VEGFR2) on brain endothelial cells, activating Src family kinases and downstream signaling cascades that disrupt TJ proteins including Claudin‐5, occludin, and ZO‐1 [119, 121]. VEGF also acts on astrocytes, further upregulating AQP4 and promoting end‐foot swelling [122]. The resulting end‐foot edema physically compresses the microvascular lumen, reduces capillary perfusion, and exacerbates BBB hyperpermeability [123].

In addition, ischemia or mechanical trauma activates MMP‐9 in astrocytes and endothelial cells. MMP‐9 degrades the vascular basement membrane and cleaves TJ proteins, directly increasing paracellular leakage [124, 125]. Proinflammatory cytokines such as IL‐1β and TNF‐α, released from activated microglia, further upregulate astrocytic AQP4 and suppress glutamate transporter‐1 (GLT‐1) and glutamate–aspartate transporter (GLAST) on astrocyte end‐feet [126, 127, 128, 129, 130]. Glutamate accumulation leads to excitotoxic injury to neurons and end‐feet, perpetuating a vicious cycle of neuroinflammation and barrier breakdown [131]. Collectively, reactive astrogliosis with AQP4 upregulation and mislocalization, VEGF‐mediated junctional disruption, MMP‐9‐dependent basement membrane degradation, and glutamate excitotoxicity converge to dismantle astrocyte end‐foot support and BBB integrity in both IS and TBI.

Overall, BBB disruption in both IS and TBI arises from endothelial TJ degradation, enhanced caveolar transcytosis, and multiple cell death pathways, together with pericyte detachment and loss, as well as astrocyte end‐foot dysfunction characterized by AQP4 mislocalization and VEGF‐driven barrier damage. Collectively, these processes compromise vascular integrity, thereby facilitating peripheral immune cell infiltration and sustained glial activation, which then trigger the neuroinflammatory cascades detailed in the following section (Figure 1).

**FIGURE 1:**
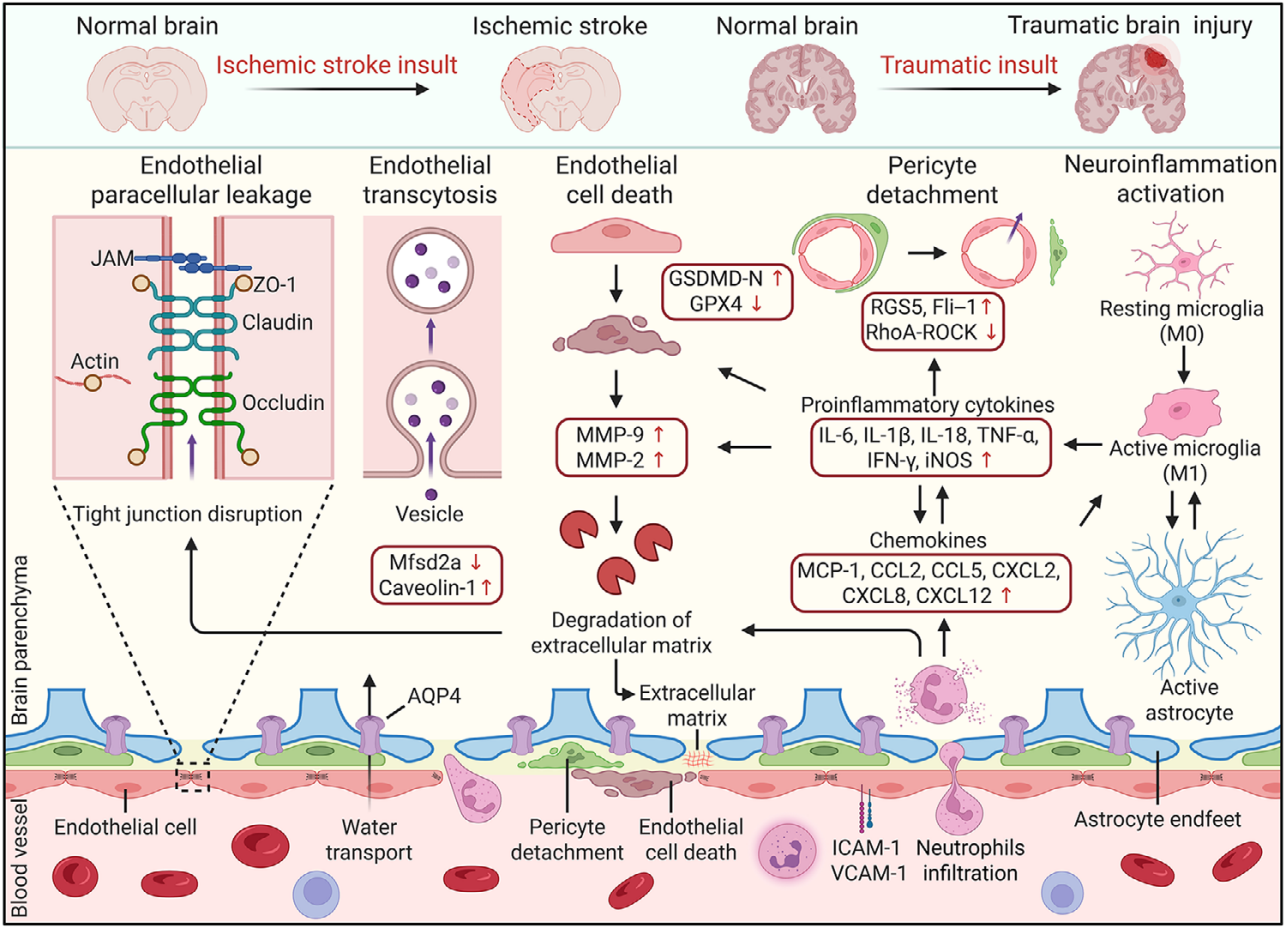
Schematic overview of BBB disruption and secondary brain injury after IS and TBI. Both conditions trigger endothelial cell dysfunction, pericyte contraction, and upregulated AQP4 expression in astrocytic end‐feet. These changes compromise barrier integrity, facilitating peripheral immune cell infiltration into the brain parenchyma. Concurrently, resident microglia and astrocytes become activated and release proinflammatory cytokines, which further amplify BBB damage and propagate secondary brain injury cascades.

### 3.2 Neuroinflammation

After BBB breakdown in IS and TBI, resident microglia and astrocytes become activated and release chemokines that recruit peripheral immune cells across the compromised barrier. The ensuing infiltration of neutrophils, monocytes, and T cells, together with sustained glial activation, drives a self‐amplifying neuroinflammatory cascade that exacerbates secondary injury.

#### 3.2.1 Microglial M1/M2 Polarization

IS triggers rapid activation of resident microglia in the ischemic core and penumbra within minutes to hours after arterial occlusion [132]. In the mouse MCAO model, M1 proinflammatory markers including CD86, iNOS, IL‐6, and TNF‐α are significantly elevated, peaking at 1–3 days after reperfusion, and correlate with infarct expansion [133, 134, 135]. In contrast, M2 anti‐inflammatory markers such as CD206, Arg‐1, IL‐10, and TGF‐β are markedly decreased during the acute phase, with partial recovery observed predominantly in the peri‐infarct region during the subacute phase, reflecting an unbalanced M1/M2 polarization [136, 137, 138]. Importantly, microglial responses exhibit marked region‐specific heterogeneity, with microglia largely absent in the necrotic core where infiltrating macrophages take over debris clearance, while microglia amass at the peri‐infarct periphery adopting proinflammatory profiles and heightened phagocytic activity [136, 139]. The M1/M2 polarization balance is governed by the PI3K/Akt/NF‐κB and PASK–EEF1A1 pathways [138]. TLR4/MyD88 signaling drives NF‐κB‐dependent M1 polarization, whereas PPAR‐γ activation shifts the balance toward M2 and reduces infarct volume via inhibition of ERK and activation of Akt signaling [140, 141, 142].

After primary mechanical injury in TBI, BBB disruption and release of DAMPs activate resident microglia. Activated microglia change morphology, migrate to the lesion, and can polarize into either proinflammatory M1 or anti‐inflammatory M2 phenotypes [143]. TBI induces even more rapid microglial activation, often within minutes after mechanical impact [39]. In the CCI model, M1 markers including CD86, TNF‐α, and IL‐6 are detectable at 3–6 h, peak at 1–3 days, and persist for up to 2 weeks in the perilesional cortex, a critical region where infiltrating immune cells interact with resident microglia to shape the local inflammatory milieu [39]. However, M2 markers such as CD206, IL‐10, and Arg‐1 are not significantly elevated after TBI during acute phase [144]. Injury severity dictates moderate‐to‐severe TBI results in sustained M1 activation, which correlates with chronic neuroinflammation and cognitive deficits [39]. STING, MAPK, JAK/STAT, and NF‐κB pathways regulate microglial polarization in TBI, but mechanical disruption of the BBB allows early infiltration of peripheral macrophages, which further shape the local inflammatory milieu [145, 146, 147, 148].

#### 3.2.2 Astrocyte Reactivity and A1/A2 Polarization

Reactive astrogliosis is prominent in the ischemic penumbra in IS [149]. Using transcriptomic profiling, ischemia‐induced reactive astrocytes predominantly assume neurotoxic A1 phenotype, driven by microglial‐derived IL‐1α, TNF, and C1q [150]. A1 astrocytes lose their glutamate transporter GLT‐1 and upregulate complement C3, contributing to neuroinflammation. Foundational studies have established that this A1 polarization is triggered by activated microglia through the synergistic secretion of IL‐1α, TNF, and C1q, with all three cytokines being both necessary and sufficient to induce the A1 phenotype [151]. Beyond the loss of GLT‐1, A1 astrocytes exhibit broader functional deficits, including impaired phagocytic capacity for synapses and myelin debris, reduced expression of synaptogenic factors, and loss of the ability to promote neuronal survival and neurite outgrowth, while acquiring a neurotoxic phenotype that induces death of neurons and mature oligodendrocytes [151]. The released glutamate overactivates glutamate receptors and mediates excitotoxicity, which elevates intracellular sodium and calcium levels, leading to mitochondrial dysfunction, protease activation, ROS accumulation, and nitric oxide release. These processes not only cause neuronal death but also further amplify neuroinflammation, creating a vicious cycle that exacerbates poststroke brain injury [152]. During the early stage of IS, microglial activation and A1 astrocyte polarization engage in a vicious cycle that exacerbates neuroinflammatory injury [153]. A2 astrocytes positive for S100A10 are observed mainly in the peri‐infarct region during the subacute phase and express neurotrophic factors such as BDNF and GDNF [154, 155]. In IS, activation of the NF‐κB pathway promotes astrocyte differentiation toward the proinflammatory A1 phenotype, thereby exacerbating neuroinflammation [156]. Additionally, the HIF‐1α/LDHA axis regulates A1/A2 astrocyte differentiation and modulates glial scar formation after cerebral ischemia [157]. In contrast, activation of the Akt–mTOR and KLF4 positively regulates the protective A2 astrocyte phenotype [152, 154], and activation of the PINK1/Parkin pathway also promotes A2 polarization following cerebral ischemia [158].

Turning to TBI, reactive astrogliosis is a hallmark of TBI, with A1 astrocytes dominating the perilesional cortex and hippocampus [159, 160]. The induction is driven by microglial‐derived IL‐1α, TNF, and C1q, which are released in large quantities due to extensive cell death [161]. A1 astrocytes are characterized by high expression of complement components, notably C3, along with proinflammatory mediators such as CXCL10, IL‐6, and IL‐1β. Concurrently, they downregulate phagocytic receptors including Mertk and Megf10, as well as synaptogenic factors like Gpc6 and Sparcl1. This phenotypic shift leads to a loss of homeostatic functions and impaired synaptic support, while promoting the secretion of neurotoxic factors that drive both neuronal and oligodendrocyte death. Moreover, A1 astrocytes amplify neuroinflammation through positive feedback loops with microglia and contribute to glial scar maturation, thereby hindering axonal regeneration and functional recovery [162]. Following TBI, A2 astrocytes distribute around the injury site where inflammation resolves. Neuroprotective functions include upregulation of BDNF and NGF, secretion of anti‐inflammatory mediators IL‐10, IGF‐1, and TGF‐β, and maintenance of glutamate and ion homeostasis. A2 astrocytes express S100A10 and PTX3 without complement component C3. Polarization toward this reparative phenotype is driven by IL‐4 and IL‐13 via the STAT6 pathway, limiting secondary damage and promoting neurological recovery after TBI [162]. Several signaling pathways regulate A1/A2 astrocyte polarization after TBI. The TLR4/NF‐κB, JAK/STAT3, MAPK, and METTL5/c‐Myc/PD‐L1 pathways promote A1 polarization and neuroinflammation [162, 163]. Conversely, PPAR‐γ activation suppresses A1 polarization, reduces inflammatory cytokine production and astrocyte activation, thereby exerting neuroprotection [164].

#### 3.2.3 Immune Infiltration

Following IS, peripheral immune cells infiltrate the brain parenchyma through disrupted BBB and fuel neuroinflammation via coordinated molecular cascades. Neutrophils are the first to arrive, driven by CXCL1/CXCR2 and CXCL2/CXCR2 chemotactic axes. Within the ischemic microenvironment, neutrophils undergo NETosis, releasing NETs. These NETs are phagocytosed by microglia, where they activate the AIM2 inflammasome, leading to Caspase‐1 cleavage and GSDMD pore formation. This process triggers pyroptosis and the release of IL‐1β and IL‐18, which amplify local inflammation [165]. Simultaneously, infiltrating Ly6C^+^CCR2^+^ monocytes respond to microglia‐derived CCL2 and differentiate into monocyte‐derived cells (MdCs). These MdCs initially adopt a CD45ʰ^i^ ^g^ʰ phagocytic phenotype that clears debris, but later shift toward a CD45ˡ°ʷ proinflammatory phenotype through NF‐κB and STAT1/3 activation, producing TNF‐α, IL‐6, and iNOS. Bidirectional crosstalk between MdCs and microglia via IL‐6/STAT3 signaling drives the emergence of repair‐associated microglia, whereas loss of this signaling aggravates fibrosis and vascular damage [166].

Beyond these myeloid‐driven responses, the S1P/S1PR signaling axis serves as a central mechanistic pathway governing lymphocyte egress from secondary lymphoid organs and subsequent recruitment to the ischemic brain. Following stroke, a steep S1P gradient is established from the spleen toward the brain, providing the chemotactic drive for lymphocyte egress [167]. S1PR1 plays a critical role in this process, as S1PR1^+^ cells constitute the predominant population deserting the spleen, and S1PR1 signaling is essential for T cell egress from secondary lymphoid organs along the S1P gradient. Concurrently, T helper and regulatory T cells are recruited to the ipsilateral hemisphere, a process associated with differential regulation of cerebral S1PR expression patterns. Notably, S1P levels are reduced in the peri‐infarct cortex while remaining high in the ischemic core, creating a gradient that guides T cell trafficking toward the injured region, and S1PR2 and S1PR3 are differentially upregulated in the ischemic hemisphere, potentially modulating the local inflammatory milieu [167]. Meanwhile, γδ T cells are recruited by CXCL10/CXCR3 and CXCL12/CXCR4 gradients. Upon arrival, γδ T cells become the predominant early source of IL‐17A, which acts on IL‐17RA expressed on neutrophils and microglia, and synergizes with TNF‐α to activate the NF‐κB and MAPK pathways, further sustaining neutrophil infiltration and microglial M1 polarization [168]. These interconnected molecular events create a self‐reinforcing inflammatory loop that expands the lesion and worsens neurological outcome.

Following TBI, BBB disruption enables swift infiltration of peripheral immune cells into the brain parenchyma. Within the injured hemisphere, monocyte‐derived macrophages (MDMs) enter the cortex within days and, contrary to prior belief, persist for at least 8 months postinjury. These long‐lived brain MDMs retain a transcriptomic signature distinct from resident microglia and are enriched for pathways linked to aging, senescence, and neurodegeneration [169]. Within the lesioned brain, MDMs actively phagocytose debris but also sustain a proinflammatory milieu through continuous expression of NOX2 and NLRP3 inflammasome components. Mechanistically, NOX2‐derived ROS in the injured brain serve as a priming signal for NLRP3 inflammasome activation, leading to Caspase‐1 cleavage and IL‐1β/IL‐18 release, a process amplified in both brain‐infiltrating neutrophils and inflammatory monocytes [170]. Furthermore, adaptive immune responses are engaged within the brain after TBI. Clonal expansion of immunoglobulin heavy and light chains is detected in blood as early as Day 9, accompanied by decreased T‐cell receptor diversity and elevated IL‐1R expression on CD4^+^ and CD8^+^ T cells in the injured hemisphere, indicating bidirectional crosstalk between brain‐infiltrating myeloid cells and T lymphocytes [171]. Together, these coordinated innate and adaptive immune responses within the central nervous system transform the acute injury into a persisting neuroinflammatory state, offering multiple therapeutic windows for intervention.

In brief, post‐TBI neuroinflammation arises from three interconnected cellular compartments, specifically microglia polarized toward a proinflammatory M1 phenotype, astrocytes skewed to the neurotoxic A1 subtype, and infiltrating peripheral immune cells including neutrophils, monocytes, and γδ T cells. Their coordinated activation amplifies cytokine release, oxidative stress, and inflammasome signaling. This sustained inflammatory milieu directly primes neurons for various forms of programmed cell death, which will be discussed in the following section (Figure 2).

**FIGURE 2:**
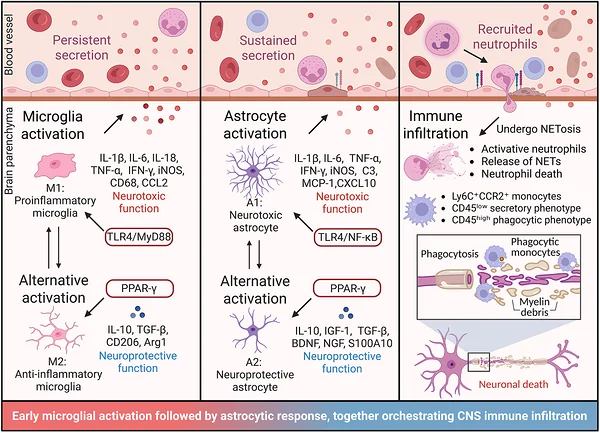
Schematic illustration of neuroinflammatory cascades following IS and TBI. After injury, microglia are activated and polarize toward the proinflammatory M1 phenotype, while astrocytes shift to the neurotoxic A1 phenotype. Both cell types release inflammatory cytokines and chemokines, which promote peripheral immune cell recruitment across the disrupted BBB into the central nervous system. Once infiltrated, these immune cells further differentiate and amplify neuroinflammation, thereby exacerbating secondary brain injury.

### 3.3 Neuronal Death, Excitotoxicity, and Oxidative Stress

Building upon the sustained neuroinflammatory environment discussed above, the following section addresses the downstream molecular events that directly compromise neuronal survival. In both IS and TBI, the convergence of inflammatory mediators, metabolic stress, and ionic dysregulation triggers a series of cell death programs, including apoptosis, necroptosis, pyroptosis, and ferroptosis [11, 172]. Concurrently, excessive glutamate release and impaired uptake drive excitotoxic injury, while mitochondrial dysfunction and NADPH oxidase (NOX) activation produce overwhelming ROS and reactive nitrogen species (RNS). These interconnected mechanisms amplify each other, ultimately leading to widespread neuronal loss and neurological deterioration. The current section will dissect each of these terminal pathways and their relevance to secondary brain injury.

#### 3.3.1 Apoptosis, Necroptosis, Pyroptosis, and Ferroptosis

Neuronal apoptosis in both IS and TBI is executed through the intrinsic mitochondrial pathway as well as the extrinsic death receptor pathway [173, 174]. These two pathways converge on the activation of executioner Caspase‐3, which drives irreversible neuronal death. The intrinsic pathway is triggered by mitochondrial stress. In IS, excessive Ca^2^^+^ influx through overactivated N‐methyl‐D‐aspartate receptors (NMDARs) activates calpain, which cleaves BID into tBID. tBID promotes Bax oligomerization and mitochondrial outer membrane permeabilization, leading to cytochrome *c* release [175]. In TBI, mechanical impact and subsequent secondary cascades similarly cause mitochondrial dysfunction and cytochrome *c* release [173]. Once in the cytosol, cytochrome *c* binds to apoptotic protease‐activating factor‐1 and Procaspase‐9 to form the apoptosome. This complex induces Caspase‐9 dimerization and autoactivation. Active Caspase‐9 then cleaves and activates Caspase‐3, initiating intrinsic apoptosis [176].

The extrinsic pathway is activated by death ligands. In both IS and TBI, elevated TNF‐α, Fas ligand FASL, and TNF‐related apoptosis‐inducing ligand (TRAIL) bind to their respective death receptors including TNF receptor‐1 (TNF‐R1), Fas, and TRAIL receptor. Receptor ligation recruits the adaptor protein TRADD and subsequently FADD, which facilitates Procaspase‐8 dimerization and activation [177]. Active Caspase‐8 directly cleaves Caspase‐3. Caspase‐8 can also amplify the mitochondrial pathway by cleaving BID into tBID, linking the extrinsic and intrinsic cascades [178]. Thus, despite their distinct primary insults, IS and TBI share the same core apoptotic machinery. The intrinsic pathway is dominated by mitochondrial cytochrome *c* release and apoptosome‐mediated Caspase‐9 activation, while the extrinsic pathway relies on death receptor‐mediated Caspase‐8 activation. Both pathways ultimately activate Caspase‐3, which cleaves downstream substrates such as inhibitor of Caspase‐activated DNase and gelsolin, leading to DNA fragmentation, cytoskeletal breakdown, and neuronal apoptosis [173].

Necroptosis is a caspase‐independent regulated necrosis that contributes to secondary brain damage after IS and TBI [179, 180]. Its core pathway is initiated by TNF‐α binding to TNF‐R1. This triggers membrane bound Complex I containing TRADD, RIPK1, TRAF2/5, cIAP1/2, and LUBAC. Within Complex I, RIPK1 is polyubiquitinated to promote cell survival via NF‐κB and MAPK signaling. Deubiquitination of RIPK1 by CYLD or cIAP inhibition causes RIPK1 to dissociate from TNF‐R1 and form cytosolic Complex II with TRADD, FADD, and Procaspase‐8 [172]. When Caspase‐8 activity is blocked, Complex II converts into the necrosome Complex IIc. The necrosome features RHIM‐dependent oligomerization of RIPK1 and RIPK3, leading to their auto and cross phosphorylation. RIPK3 then phosphorylates MLKL. Phosphorylated MLKL oligomerizes and translocates to the plasma membrane, forming pore like clusters. This disrupts membrane integrity, causing cell swelling and lytic death with release of DAMPs [181]. In IS, necroptosis is activated in the penumbra after reperfusion, driven by oxidative stress and TNF‐α from microglia [182]. In TBI, mechanical injury rapidly induces TNF‐α and death ligands, leading to necrosome assembly in neurons, astrocytes, and microglia [183, 184]. The core TNF‐R1–RIPK1–RIPK3–MLKL machinery is conserved between both conditions, and its activation amplifies neuroinflammation and secondary injury.

Pyroptosis is a proinflammatory regulated cell death mediated by GSDMD pore formation [185]. In both IS and TBI, pyroptosis contributes to neuronal loss through activation of the NLRP3 inflammasome pathway [186, 187]. In IS, cerebral ischemia and reperfusion induce mitochondrial ROS accumulation. ROS activate the NLRP3 inflammasome, leading to recruitment of ASC and Procaspase‐1. Autocleavage produces active Caspase‐1, which cleaves GSDMD into its N‐terminal fragment GSDMD‐N. GSDMD‐N oligomerizes to form membrane pores, causing cell swelling, lysis, and release of IL‐1β and IL‐18. These cytokines amplify neuroinflammation and directly promote neuronal excitotoxicity. Caspase‐1 also processes pro‐IL‐1β and pro‐IL‐18 into their mature forms, sustaining a vicious cycle of inflammation and neuronal injury [188]. In TBI, mechanical injury triggers rapid NLRP3 inflammasome activation in microglia, astrocytes, and neurons. Additionally, the noncanonical pathway involving Caspase‐11 is engaged. Lipopolysaccharide leaking through the disrupted BBB activates Caspase‐11, which directly cleaves GSDMD independent of NLRP3 [189, 190]. Both canonical Caspase‐1 and noncanonical Caspase‐11 converge on GSDMD cleavage, leading to pyroptotic cell death. The resultant IL‐1β and IL‐18 release exacerbates neuroinflammation, disrupts synaptic integrity, and drives secondary neuronal degeneration. The core molecular axis in both conditions is NLRP3 ASC Caspase‐1 GSDMD for the canonical pathway and Caspase‐11 GSDMD for the noncanonical pathway. Pyroptosis thus represents a common terminal mechanism linking initial ischemic or traumatic insult to neuronal death in IS and TBI.

Ferroptosis is an iron‐dependent regulated cell death driven by lipid peroxidation [191]. In both IS and TBI, ferroptosis contributes to neuronal death through iron dysregulation, glutathione GSH depletion, and GPX4 inactivation [192, 193, 194]. In IS, ischemia–reperfusion induces ROS accumulation and BBB disruption. Excess ferrous iron Fe^2^^+^ accumulates via transferrin receptor‐1 upregulation and ferritinophagy mediated by nuclear receptor coactivator‐4 (NCOA4). Fe^2^^+^ drives the Fenton reaction to generate hydroxyl radicals that oxidize polyunsaturated fatty acids PUFAs. Acyl CoA synthetase long chain family member‐4 (ACSL4) incorporates PUFAs into membranes, while lipoxygenases LOX promote lipid peroxidation. Simultaneously, reduced cystine glutamate antiporter SLC7A11 function depletes GSH, inactivating GPX4 and allowing lipid peroxide accumulation, leading to membrane rupture and ferroptosis [195, 196]. In TBI, mechanical impact causes immediate hemorrhage and iron overload. Fe^2^^+^ catalyzes Fenton chemistry, producing ROS that overwhelm antioxidant defenses. TBI also reduces GPX4 activity and GSH levels due to system Xc^−^ inhibition by excess extracellular glutamate. ACSL4 and 12/15 LOX are activated, amplifying lipid peroxidation [197, 198]. Unlike IS, TBI induced ferroptosis develops rapidly from hemorrhagic and mechanical injury, with persistent iron deposition [199]. The core molecular axis in both conditions includes TfR1, DMT1, NCOA4, ACSL4, LOX, SLC7A11, GSH, and GPX4. Ferroptosis thus represents a convergent mechanism linking iron overload and oxidative stress to neuronal death in IS and TBI.

#### 3.3.2 Glutamate Excitotoxicity

Glutamate excitotoxicity is a central mechanism driving neuronal death in both IS and TBI [200, 201]. It results from excessive glutamate accumulation in the extracellular space, leading to overactivation of ionotropic glutamate receptors and downstream prodeath signaling [202].

Cerebral blood flow reduction causes ATP depletion and failure of Na^+^/K^+^ ATPase after IS. Membrane depolarization reverses Na^+^‐dependent glutamate transporters EAATs, switching them from uptake to release mode [203, 204]. Extracellular glutamate concentration rapidly increases from basal low micromolar levels to a markedly elevated level within minutes [205, 206]. This excess glutamate hyperactivates NMDARs and α‐amino‐3‐hydroxy‐5‐methy‐4‐isoxazolepropionic acid receptors AMPARs on postsynaptic neurons. NMDAR overactivation triggers massive Ca^2^^+^ influx. Cytosolic Ca^2^^+^ overload activates calpain, which cleaves cytoskeletal proteins and converts BID to tBID, promoting mitochondrial outer membrane permeabilization [203]. Ca^2^^+^ also activates nNOS, producing nitric oxide that reacts with superoxide to form peroxynitrite. Peroxynitrite damages proteins, lipids, and DNA, as well as inhibits mitochondrial respiration. Mitochondrial Ca^2^^+^ overload opens the mitochondrial permeability transition pore (mPTP), dissipating membrane potential, halting ATP synthesis, and releasing cytochrome *c* and apoptosis inducing factor (AIF) [207]. These events converge on caspase‐dependent apoptosis and caspase‐independent parthanatos. Additionally, sustained NMDAR activation recruits death‐associated protein kinase‐1 to the NR2B subunit, phosphorylating NR2B at Ser1303 to further increase channel activity. The NR2B–PSD95–nNOS complex couples receptor activation to sustained NO production, amplifying oxidative stress [203].

In TBI, mechanical impact directly stretches and tears neuronal membranes, causing immediate, nonvesicular glutamate release from injured cells [208]. Primary injury also disrupts the BBB, allowing serum glutamate to enter brain parenchyma [209]. Similar to IS, ATP depletion and ionic dysregulation impair astrocytic glutamate transporters GLAST and GLT‐1, reducing uptake and sustaining high glutamate levels [130, 210]. Excess glutamate overactivates NMDARs, triggering massive Ca^2^^+^ influx. Intracellular Ca^2^^+^ overload activates calpain, which cleaves cytoskeletal proteins, and neuronal nitric oxide synthase nNOS, producing peroxynitrite [211]. Mitochondrial Ca^2^^+^ overload opens the mPTP, dissipating membrane potential, depleting ATP, and releasing cytochrome *c* and AIF [212]. These events converge on caspase‐dependent and caspase‐independent neuronal death. ROS further inhibit glutamate transporters, creating a self‐sustaining excitotoxic cycle that expands secondary brain damage and promotes neuronal death [213].

In both pathologies, the final common executioners include calpain‐mediated cytoskeletal degradation, nNOS‐derived peroxynitrite, mPTP opening, and Caspase‐3 activation [214]. Key differences lie in the initiating events. IS relies on energy failure and transporter reversal, whereas TBI adds mechanical membrane disruption and immediate channel‐mediated release [208, 215]. The temporal profile also diverges. IS excitotoxicity peaks during reperfusion, while TBI excitotoxicity peaks within hours and can persist for days due to sustained GLT‐1 loss [210, 216, 217]. However, the core molecular axis of NMDAR Ca^2^^+^ calpain nNOS mitochondrial dysfunction is shared, making glutamate excitotoxicity a convergent mechanism of neuronal death in IS and TBI.

#### 3.3.3 Oxidative Stress

Oxidative stress is a central driver of neuronal death in both IS and TBI, a consequence of excessive ROS and RNS [218, 219]. The high oxygen consumption of brain, abundant polyunsaturated fatty acids, and weak antioxidant defenses render it exceptionally vulnerable.

In IS, ischemia–reperfusion impairs the mitochondrial electron transport chain, superoxide anion overproduction follows. ROS directly oxidize lipids, generating 4‐hydroxy‐2‐nonenal 4‐HNE, and damage proteins and DNA [220]. Lipid peroxidation disrupts membrane integrity and inactivates ion pumps. ROS also deplete glutathione and suppress GPX4, crippling endogenous antioxidant capacity [221]. Simultaneously, ROS activate the NLRP3 inflammasome and NF‐κB, driving interleukin‐1β and TNF‐α release. These inflammatory mediators further stimulate NOXs in microglia, amplifying ROS production [222, 223]. The resulting oxidative injury opens the mPTP, releases cytochrome *c*, and triggers caspase‐dependent apoptosis [224]. Free iron, derived from hemoglobin and ferritin, catalyzes Fenton chemistry, producing hydroxyl radicals that intensify lipid peroxidation and ferroptosis [225].

The temporal evolution of oxidative stress after IS follows a distinct triphasic pattern. In the early phase, occurring within minutes to hours after reperfusion, mitochondrial electron transport chain impairment drives a rapid burst of superoxide production, followed by a second phase mediated by xanthine oxidase activation. During reperfusion, a third phase dominated by NOX activation occurs, generating sustained ROS production that amplifies oxidative injury [226, 227]. Spatially, the ischemic core exhibits severe and persistent oxidative damage due to profound energy failure, mitochondrial dysfunction, and iron release from hemorrhagic components, whereas the peri‐infarct region shows more moderate but sustained oxidative stress that contributes to ongoing neuronal injury and inflammatory cell activation through a self‐amplifying loop linking oxidative damage with innate immune responses [227, 228].

In TBI, mechanical impact directly disrupts mitochondria and activates NOX2, causing a rapid ROS burst [229]. Extravasated blood introduces fibrinogen and iron. Fibrinogen, acting via the CD11b integrin receptor, upregulates NOX2 and iNOS in microglia and macrophages, generating both superoxide and nitric oxide [230, 231]. These combine to form peroxynitrite, a potent RNS that nitrates tyrosine residues, damages DNA, and inhibits mitochondrial respiration. Iron drives hydroxyl radical formation and lipid peroxidation, marked by elevated malondialdehyde MDA and 4‐HNE [232]. Oxidative stress inactivates glutamate transporters GLT‐1, leading to glutamate accumulation and excitotoxicity [128]. Excitotoxic Ca^2^^+^ influx further increases mitochondrial ROS. The downstream effects include calpain‐mediated cytoskeletal breakdown, PSD95 nitration, synaptic loss, and opening of the permeability transition pore, releasing AIF and causing caspase‐independent parthanatos [233, 234]. In both conditions, mitochondrial complexes I and III, NOXs, and xanthine oxidase are the primary ROS sources [235, 236]. iNOS and nNOS are the main RNS sources. Antioxidant enzymes such as superoxide dismutase, catalase, and GPX4 are downregulated or inactivated, tipping the balance toward oxidative injury. Thus, oxidative stress acts as a convergent mechanism, linking ischemia–reperfusion or mechanical trauma to neuronal death in IS and TBI.

The temporal and spatial profiles of oxidative stress in TBI exhibit distinct characteristics that differ from those in IS. Within minutes to hours after mechanical impact, an immediate ROS burst occurs, driven by mitochondrial disruption, rapid NOX2 activation, and the release of free iron from hemoglobin and intracellular storage sites due to localized acidosis, which together catalyze hydroxyl radical formation via the Fenton reaction [237, 238]. This early phase is followed by a sustained oxidative stress phase lasting hours to days, characterized by progressive lipid peroxidation with elevated malondialdehyde and 4‐hydroxynonenal, protein carbonylation, 3‐nitrotyrosine formation, and progressive depletion of endogenous antioxidants such as glutathione and superoxide dismutase [237, 238]. Spatially, the contusion core exhibits the most severe and immediate oxidative injury, while oxidative stress propagates to perilesional areas through neuroinflammation‐driven ROS production by activated microglia and infiltrating immune cells, as well as diffusion of lipid peroxidation products, ultimately contributing to secondary tissue damage and synaptic dysfunction [237, 238].

In summary, neuronal death after IS and TBI arises from an interconnected network of programmed cell death, excitotoxicity, and oxidative stress. These processes mutually reinforce one another, creating a vicious cycle that amplifies secondary injury and explains why single‐target interventions have largely failed clinically. The following part reviews emerging therapeutic strategies that directly target these terminal cascades (Figure 3).

**FIGURE 3:**
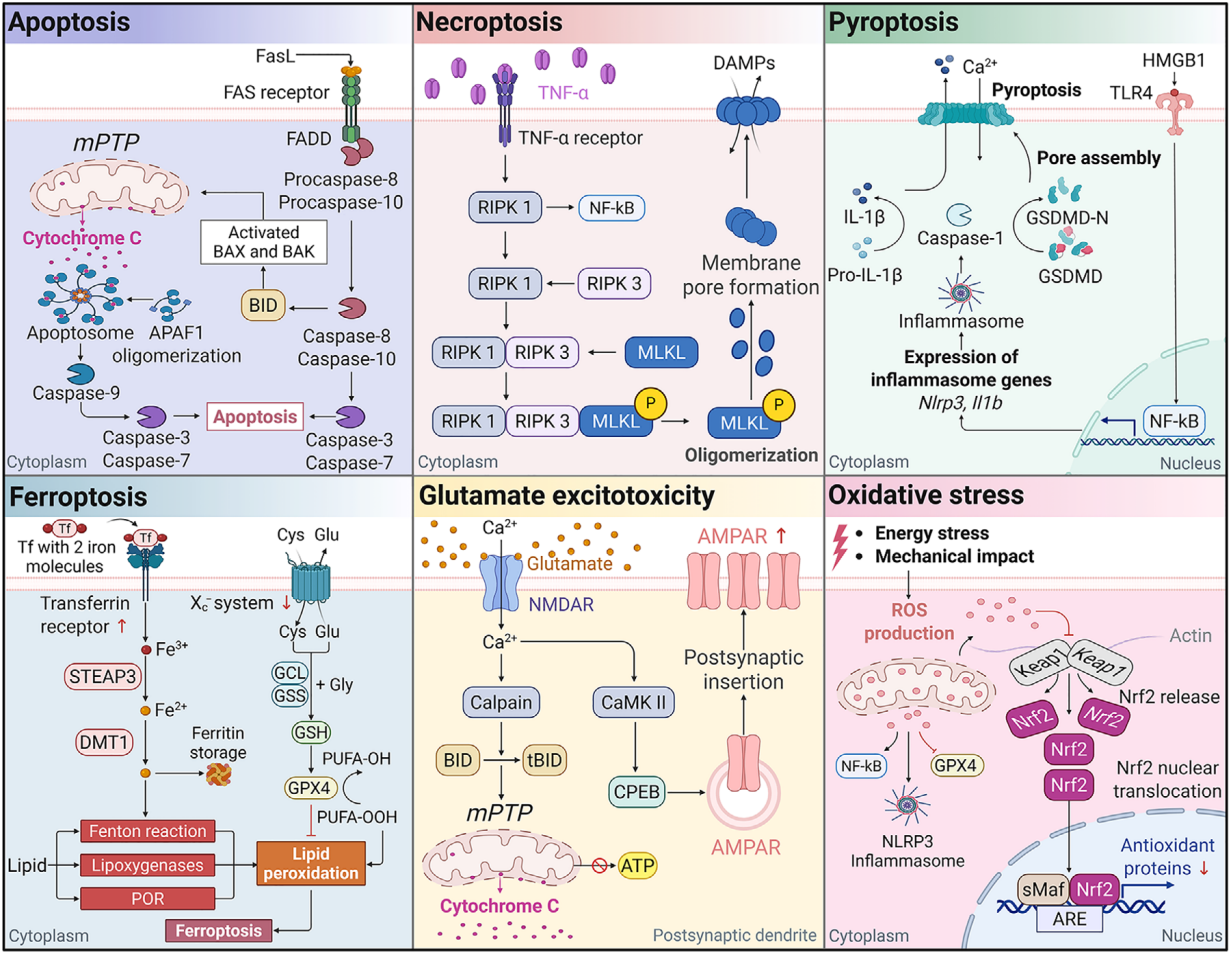
Molecular mechanisms of neuronal death and injury following IS and TBI. Both conditions trigger multiple programmed cell death pathways, including apoptosis, necroptosis, pyroptosis, and ferroptosis. Concurrently, oxidative stress and glutamate‐mediated excitotoxicity are markedly enhanced. Together, these interconnected processes drive widespread neuronal loss and aggravate secondary brain injury after IS and TBI.

## 4 Emerging Therapeutic Strategies

Given the interconnected cascades of BBB disruption, neuroinflammation, and neuronal death shared by IS and TBI, targeting these pathways has become a major focus of translational research. The following sections summarize promising therapeutic approaches that have recently advanced into preclinical studies or clinical trials. Specifically, we discuss emerging interventions aimed at preserving BBB integrity, modulating neuroinflammatory responses, and inhibiting terminal cell death pathways, with a focus on parallel evaluation across both disorders.

### 4.1 Therapeutic Approaches Targeting BBB Disruption

#### 4.1.1 Targeting Endothelial Cell Dysfunction

For IS, several emerging therapies directly target TJ disruption to preserve BBB integrity. Disruption of the ROCK1/MLC/NMMHC IIA‐actin positive feedback loop, using the NMMHC IIA inhibitor blebbistatin, the ROCK1 inhibitor Y‐27632, or the actin depolymerizer cytochalasin D, significantly reduces TJ protein degradation and alleviates cerebral ischemia–reperfusion‐induced BBB disruption [83]. Danhong injection protects against BBB TJ disruption by activating the Wnt/β‐catenin pathway, thereby upregulating Claudin‐5, occludin, and ZO‐1 while suppressing MMP‐9 expression [239]. Luteolin counteracts BBB TJ disruption after IS by inhibiting LRP1 and subsequently reducing MMP‐9 expression, which prevents the degradation of Claudin‐5, occludin, and ZO‐1 [74]. Beyond MMP‐9 inhibition, trifluoperazine acts on the CaM/MLCK pathway to reduce endothelial retraction and improve TJ integrity [240]. Rosmarinic acid serves as another novel agent that protects against ischemia/reperfusion‐induced TJ disruption by directly binding to MMP‐9 and MMP‐2, thereby upregulating ZO‐1, occludin, and Claudin‐5 while suppressing MMP‐9/MMP‐2 expression [241]. In TBI, similar TJ‐directed strategies are emerging. Thyroid hormone counteracts TBI‐induced TJ disruption by upregulating ZO‐1 expression, downregulating MMP‐9 and TLR‐4, and promoting A2 astrocyte activity, thereby preserving BBB integrity [71]. Inhibiting endothelial S1PR2, using the selective antagonist JTE‐013 or shRNA, preserves BBB TJ integrity after TBI by restoring occludin and ZO‐1 expression via activation of the PI3K–Akt pathway [242]. Fucoxanthin, a marine carotenoid, protects against TBI‐induced TJ disruption by activating mitophagy, thereby preserving TJ and adherens junction proteins, inhibiting MMP‐9 expression, and reducing endothelial cell apoptosis and ferroptosis [243]. Notably, inhibition of Transglutaminase 2 alleviates TBI‐induced TJ disruption by suppressing the IL‐17/MMP‐9 axis, which restores Claudin‐5 and ZO‐1 expression and preserves BBB integrity [75]. Collectively, these emerging interventions highlight that directly targeting endothelial TJ damage represents a promising therapeutic avenue to preserve BBB integrity after both IS and TBI.

Emerging therapeutic strategies are also targeting the pathological increase in caveolae‐mediated transcytosis, an early and dominant mechanism of BBB breakdown in both IS and TBI. In IS, targeting GATA zinc finger domain‐containing 1 in cerebral endothelial cells alleviates BBB dysfunction by downregulating CD36 expression and inhibiting caveolae‐mediated transcytosis, thereby reducing infarct volume and improving neurological outcomes [86]. Glial growth factor 2 alleviates IS‐induced BBB disruption by reversing occludin degradation and inhibiting both Mfsd2a/Cav‐1‐mediated transcytosis and Pdlim5/YAP/TAZ‐mediated paracellular permeability [244]. L‐borneolum and its main components reduce BBB dysfunction after IS by modulating the Mfsd2a/Cav‐1 signaling axis, thereby decreasing caveolae‐mediated transcytosis and improving barrier integrity [245]. In TBI, upregulation of Cav‐1 and downregulation of Mfsd2a similarly drive excessive transcytosis. Activating the Wnt/β‐catenin signaling pathway with the agonist LiCl alleviates TBI‐induced BBB dysfunction by reducing Cav‐1‐mediated vesicular transcytosis and upregulating Mfsd2a expression, thereby preserving TJ integrity and improving neurological outcomes [95]. The S1PR1 modulator CYM‐5442 alleviates TBI‐induced BBB dysfunction by reducing cerebrovascular endothelial vesicular transcytosis, an effect linked to restoring Mfsd2a‐mediated S1P transport, which preserves TJ integrity and improves neurological outcomes [77]. Collectively, these emerging interventions underscore that targeting the Mfsd2a/Cav‐1 axis and downstream transcytotic machinery constitutes a promising therapeutic approach to preserve BBB integrity in both IS and TBI.

In addition to targeting TJ disassembly and pathological transcytosis, emerging therapies are also being developed to combat the direct loss of brain microvascular endothelial cells through multiple programmed cell death pathways after IS and TBI. In IS, recombinant ABI3BP preserves BBB integrity by restoring TJ expression and suppressing endothelial apoptosis via PI3K/Akt signaling [246]. Similarly, recombinant FGF17 protects against ischemia/reperfusion‐induced BBB disruption by activating the FGFR3/PI3K/Akt signaling pathway and reducing endothelial cell apoptosis [247]. Infliximab, an anti‐TNF‐α antibody, represents a novel therapeutic strategy that alleviates IS‐induced BBB TJ disruption by inhibiting endothelial necroptosis via the TNF‐α/TNFR1 pathway [85]. Targeting endothelial ferroptosis through p23, which enhances GPX4 stability, effectively protects against ischemia/reperfusion‐induced BBB injury [248]. Additionally, Se‐(methyl)‐selenocysteine reduces endothelial lipid peroxidation and upregulates TJ proteins in an Akt/GSK3β‐dependent manner [249]. In TBI, microRNA‐223 alleviates stretch‐induced endothelial apoptosis by suppressing RhoB expression, identifying miR‐223 as a potential therapeutic target [250]. Mitochondrial dysfunction and subsequent endothelial apoptosis are key drivers of BBB breakdown after blast TBI, and targeting the P2RX7 pathway to preserve mitochondrial integrity shows promise [106]. The IL‐25–ILC2–IL‐13 axis promotes BBB repair after TBI by suppressing CXCL‐10 and endothelial pyroptosis, highlighting IL‐25 as a novel therapeutic candidate [105]. Finally, the ferroptosis inhibitor Ferrostatin‐1 significantly reduces brain microvascular endothelial cell death, BBB hyperpermeability, and TJ loss in TBI models [103]. Collectively, these emerging interventions demonstrate that targeting endothelial cell death represents a promising multipronged strategy to preserve BBB integrity and limit secondary brain injury in both IS and TBI.

#### 4.1.2 Targeting Pericyte Dysfunction

Beyond endothelial cell dysfunction, emerging evidence highlights that pericyte injury, including loss, morphological abnormality, and cell death, also critically contributes to BBB breakdown after IS and TBI, and novel therapeutic strategies targeting pericytes have shown promise.

In IS, several emerging interventions protect against pericyte damage and subsequent BBB disruption. Electroacupuncture serum attenuates pericyte apoptosis and migration while enhancing pericyte viability, thereby preserving TJ integrity [251]. Overexpression of ITGA8 via adeno‐associated virus restores pericyte morphology and vascular coverage by activating the Rho–ROCK/TGF‐β1 axis [114]. Endothelial cell‐derived exosomes preserve BBB integrity by activating PDGF–PDGFRβ and Ang1/Ang2–Tie2 signaling, thus improving endothelial–pericyte communication [252]. Edaravone dexborneol protects against pericyte loss and BBB disruption through PDGFB/PDGFR‐β and vitronectin–integrin pathways [253]. Furthermore, combined therapy with fasudil and Necrostatin‐1 prevents delayed pericyte loss and preserves microcirculation perfusion by inhibiting necroptosis [254].

In TBI, novel pericyte‐targeted strategies have also emerged. Fli‐1 siRNA delivered via lateral ventricle injection mitigates pericyte loss, neuroinflammation, and BBB disruption by inhibiting Fli‐1 expression and disrupting pericyte–microglia interactions [113]. Targeting the HMGB1–S100B/RAGE axis with the RAGE inhibitor FPS‐ZM1 or genetic knockout protects hippocampal pericytes, preserves vascular basement membranes and TJ proteins, and reduces perivascular edema [255]. Inhibition of NET formation or Dectin‐1 prevents pericyte dysfunction and BBB breakdown by blocking histone–Dectin‐1‐induced CD11b upregulation in pericytes [256]. Finally, atorvastatin‐loaded PEGylated liposomes, a nanoparticle‐based therapy, mitigates pericyte apoptosis and BBB disruption by restoring Cav‐1 expression, reducing TNF‐α levels, and attenuating pericyte and astrocyte cell death [92]. Collectively, these interventions highlight that targeting pericyte dysfunction and death represents a promising multifaceted strategy to preserve BBB integrity and improve neurological outcomes after both IS and TBI.

#### 4.1.3 Targeting Astrocyte End‐Feet Dysfunction

In addition to targeting endothelial and pericyte dysfunction, emerging therapeutic strategies also focus on preserving astrocyte end‐foot integrity and restoring perivascular AQP4 polarization, which are critical for BBB maintenance and glymphatic function after both IS and TBI.

In IS, several emerging interventions alleviate astrocyte end‐foot swelling and BBB disruption by modulating AQP4 expression or polarization. SKF‐86002, a p38MAPK inhibitor, reduces cerebral edema and BBB dysfunction by suppressing astrocyte proliferation, oxidative stress, inflammation, and downregulating CX43/AQP4 expression [257]. PEG/cRGD‐modified liposome‐encapsulated emodin provides brain‐targeted delivery and inhibits AQP4 expression, thereby mitigating astrocyte end‐foot swelling and BBB disruption [258]. Astrocyte‐specific deletion of NBCe1 represents a genetic strategy that preserves perivascular AQP4 polarization, upregulates Kir4.1, and reduces reactive astrogliosis and inflammation [259]. Wuling San targets the TRPM2/NLRP3 pathway to restore perivascular AQP4 polarization, reduce brain edema, and protect TJ integrity [260].

In TBI, novel therapies also focus on restoring AQP4 polarity and astrocyte function. Transferrin receptor‐targeted nanomedicine loaded with fluvoxamine crosses the BBB and restores perivascular AQP4 polarization, preserving BBB integrity and improving glymphatic function [261]. Minocycline reduces AQP4 expression, thereby improving TJ integrity and astrocyte morphology [262]. Astragaloside IV inhibits AQP4 expression via the Akt/FoxO3a/AQP4 pathway and suppresses AQP4 subcellular localization via the TRPV4/cAMP/PKA pathway, alleviating cytotoxic edema [263]. Astrocyte‐derived exosomal miR‐211‐5p downregulates GFAP, MMP‐9, and AQP4 while maintaining TJ proteins ZO‐1 and Claudin‐5, preserving BBB integrity [125]. Collectively, these interventions highlight that targeting astrocyte end‐foot dysfunction and AQP4 polarity represents a promising therapeutic avenue to preserve BBB integrity and improve neurological outcomes after both IS and TBI.

### 4.2 Therapeutic Approaches Targeting Neuroinflammation

#### 4.2.1 Therapeutic Approaches Targeting Microglia

Disruption of the BBB after IS or TBI not only compromises vascular integrity but also initiates a robust neuroinflammatory cascade that exacerbates secondary brain injury and impedes neurological recovery. As the resident immune cells of the central nervous system, microglia play a pivotal role in this process. Upon BBB breakdown, microglia become activated and polarize toward a proinflammatory M1 phenotype, releasing cytokines and chemokines that amplify tissue damage. Therefore, emerging therapeutic strategies have increasingly focused on modulating microglial M1/M2 polarization as a means to alleviate neuroinflammation and improve outcomes.

Emerging evidence has identified a range of novel therapeutic interventions that ameliorate neuroinflammation by modulating microglial M1/M2 polarization after both IS and TBI. In IS, several agents act by suppressing the TLR4/NF‐κB pathway, a central driver of M1 activation. β‐Elemene not only inhibits TLR4/NF‐κB and MAPK signaling but also activates Akt/mTOR‐mediated autophagy, thereby shifting microglia from a proinflammatory M1 toward a reparative M2 phenotype [264]. Similarly, formononetin directly targets the TLR4/NF‐κB axis to reduce M1 polarization [265], while glibenclamide achieves comparable effects by inhibiting the Sur1–Trpm4 channel and downstream TLR4/NF‐κB signaling [266]. Beyond TLR4/NF‐κB modulation, other agents target distinct molecular hubs. Methyl isoeugenol activates Nrf2 and suppresses NLRP3 inflammasome‐mediated pyroptosis, thereby attenuating M1 responses [267]. Danhong injection suppresses ASS1 expression via the JUNB/NF‐κB pathway to promote M1‐to‐M2 conversion [268]. Tongfu‐xingshen capsule upregulates RhoA and inhibits Rock2/ASC/GSDMD‐mediated pyroptosis, restoring the M1/M2 balance [269]. Edaravone dexborneol modulates the CXCL13/CXCR5/NF‐κB axis to relieve cerebral ischemia–reperfusion injury [270]. ST909, a regulator of the cGAS/STING/IRF3 pathway, activates PI3K/Akt signaling to induce M1‐to‐M2 conversion and reduce infarct size [271]. Resveratrol activates the Epac‐1/Rap‐1 signaling pathway, which promotes M2 polarization and suppresses inflammation [272].

In TBI, similar strategies have emerged. Activating the circadian nuclear receptor Nr1d1 with SR9009 alleviates M1 polarization and improves neurological deficits [273]. β‐Asarone inhibits the Fas/FasL signaling axis, shifting microglia toward the M2 phenotype and reducing neuronal apoptosis [274]. Vitamin D acts through the TLR4/MyD88/NF‐κB pathway to favor M2 polarization and mitigate oxidative stress [275]. Finally, low‐frequency electromagnetic field exposure, a nonpharmacological intervention, promotes time‐dependent expression of both M1 and M2 markers, thereby enhancing microglial phagocytic capacity [276]. Collectively, these findings demonstrate that pharmacological and nonpharmacological strategies targeting microglial M1 polarization and the M1/M2 balance hold considerable promise for alleviating postischemic and posttraumatic neuroinflammation, and for improving functional recovery.

#### 4.2.2 Therapeutic Approaches Targeting Astrocyte

In parallel with microglia‐targeted strategies, modulating astrocytic polarization away from the neurotoxic A1 phenotype and toward the protective A2 phenotype has emerged as another critical approach to control neuroinflammation after both IS and TBI.

In IS, astrocytic KLF4 acts as a novel endogenous target that inhibits A1 activation and promotes A2 polarization via NF‐κB signaling, thereby preserving BBB integrity and improving neurological outcomes [154]. Another metabolic strategy involves promoting astrocytic glycogen mobilization, for instance, by overexpressing glycogen phosphorylase, which reduces ROS levels, suppresses NF‐κB‐mediated A1 activation, and enhances STAT3‐mediated A2 polarization, conferring neuroprotection against cerebral ischemia/reperfusion injury [277].

In TBI, esketamine alleviates A1/A2 imbalance by inhibiting the METTL5/c‐Myc/PD‐L1 signaling pathway, thereby suppressing proinflammatory A1 differentiation while promoting protective A2 polarization [163]. Pharmacological blockade of TLR4 with the novel inhibitor C34 shifts astrocytic polarization toward the anti‐inflammatory A2‐like phenotype, enhances myelin regeneration, and improves neurocognitive outcomes after TBI [278]. Additionally, remazolam regulates Connexin‐43 expression and reverses cytoskeletal damage, thereby ameliorating A1/A2 imbalance and protecting neurological function following TBI [279]. Collectively, these findings demonstrate that targeting astrocytic A1/A2 polarization through diverse molecular pathways, including transcriptional regulation, metabolic reprogramming, receptor inhibition, and gap junction modulation, offers a promising strategy to limit neuroinflammation and improve recovery after both IS and TBI.

#### 4.2.3 Therapeutic Approaches Targeting Immune Infiltration

In addition to targeting resident microglia and astrocytes, emerging therapeutic strategies are also focusing on modulating the infiltration of peripheral immune cells into the central nervous system.

In IS, targeted delivery of calcitonin gene‐related peptide to the bone marrow using aged neutrophil membrane‐coated nanoparticles promotes myeloid‐derived suppressor cell expansion and reduces myeloid cell infiltration, thereby improving neurological recovery [280]. Pharmacological reactivation of HDAC1 with compound 5104434 suppresses NF‐κB‐driven microglial proinflammatory activation and reduces T‐cell infiltration [281]. Melatonin and its macrophage membrane–biomimetic ROS–responsive nanoparticle formulation inhibit the cGAS–STING–NF‐κB pathway and block microglial NLRP3 pyroptosis, decreasing neutrophil and monocyte/macrophage infiltration [282]. Huang‐lian‐jie‐du decoction inhibits LFA‐1/ICAM‐1‐mediated adhesion and restores BBB integrity via Wnt/β‐catenin signaling, suppressing CD4^+^ T cell infiltration [283]. The small‐molecule FPR1 antagonist CP‐10 blocks NF‐κB and MAPK pathways, reducing neutrophil chemotaxis and microglial activation [132]. Platelet membrane‐coated rosiglitazone‐loaded nanoparticles polarize neutrophils toward the N2 phenotype, inhibit NETosis, and promote microglial M2 polarization via PPAR‐γ and lactate‐induced histone lactylation, shifting the microenvironment toward repair [284].

In TBI, Cl‐amidine, a selective NET inhibitor, blocks the STING‐dependent IRE1α/ASK1/JNK pathway, reducing immune cell infiltration, BBB disruption, and neuronal apoptosis [285]. The NOX2 inhibitor GSK2795039 disrupts the NOX2–ROS–NLRP3 inflammasome axis and myeloid–T cell crosstalk, attenuating infiltration of neutrophils, inflammatory monocytes, and T cells [170]. Rovelizumab, an ITGB2‐blocking antibody, inhibits neutrophil adherence, migration, and NETosis via ITGB2/ICAM‐1 disruption, reducing BBB permeability and oxidative stress [286]. Esketamine suppresses CD4^+^ T and Th17 cell infiltration and promotes M1‐to‐M2 macrophage polarization by inhibiting STAT3‐mediated glycolysis [287]. A neutralizing antibody against CXCL5 reduces inflammatory microglial/astrocytic activation and peripheral immune cell infiltration [288]. Finally, the sympatholytic agent reserpine limits immune cell mobilization from the bone marrow into the bloodstream, thereby reducing their infiltration into the brain and alleviating neuroinflammation and degeneration [289]. Collectively, these diverse pharmacological and nanomedicine‐based strategies that target peripheral immune cell trafficking offer compelling opportunities to limit neuroinflammation and improve functional recovery after both IS and TBI.

### 4.3 Therapeutic Approaches Targeting Neuronal Death, Excitotoxicity, and Oxidative Stress

#### 4.3.1 Therapeutic Approaches Targeting Neuronal Death

Neuronal apoptosis is a central pathological event in secondary brain injury following both IS and TBI, directly contributing to neurological dysfunction and poor clinical outcomes. Therefore, targeting apoptotic pathways has become a critical therapeutic strategy. In IS, 14,15‐epoxyeicosatrienoic acid activates Wnt signaling, upregulates mitophagy proteins, and inhibits the NLRP1/Caspase‐1/GSDMD pyroptosis axis, thereby reducing neuronal apoptosis and infarct volume [290]. Additionally, MiR‐495‐3p‐I upregulates canonical Wnt genes and downregulates noncanonical Wnt/MAPK genes to suppress apoptosis [291]. BMSC‐derived exosomal Egr2 activates RNF8 and promotes DAPK1 ubiquitination, increasing neuronal viability [292]. Moreover, human neural stem cell‐derived extracellular vesicles loaded with miR‐125a‐5p target IKBKG to inhibit TLR4/NF‐κB‐mediated apoptosis [293]. Silibinin activates EGFR/ERK signaling to reduce apoptosis and brain edema [294]. N‐Acetylcysteine restores the NGF‐Akt/Bad axis, reducing cleaved Caspase‐3 and cytochrome *c* levels [295]. In TBI, similarly, engineered RVG29‐conjugated nanoparticles delivering irisin promote SIRT3‐dependent mitochondrial quality control, preserving mitochondrial dynamics and reducing neuronal apoptosis [296]. The GC/I/E hydrogel reduces apoptosis while also promoting neuroprotection [297]. Zileuton blocks leukotriene production and suppresses ERK/NF‐κB/Akt pathways to lower neuronal apoptosis [298]. NDP‐MSH upregulates Merlin to reduce apoptosis and brain edema [299]. Furthermore, ZLN005 restores mitochondrial OXPHOS gene expression and reduces oxidative stress‐induced apoptosis [300]. Stigmasterol inhibits STAT3/NF‐κB/LCN‐2 signaling to suppress apoptosis [301]. Resveratrol downregulates endoplasmic reticulum stress and inhibits Caspase‐12/‐3 activation, reducing apoptosis [302]. Collectively, these diverse pharmacological, nanomedicine, and exosome‐based strategies targeting neuronal apoptosis offer promising therapeutic avenues for both IS and TBI.

Necroptosis following IS can be alleviated by several novel therapeutic strategies. The pan‐caspase inhibitor Q‐VD‐OPH, along with activation of the Nrf2/NQO1 axis or ROS scavenging, suppresses ROS‐dependent NLRP3 inflammasome activation, thereby interrupting the necroptotic cascade and reducing neuroinflammation in the ischemic penumbra [303]. Necroptosis is also effectively mitigated by stereotaxic administration of Homer1 protein into the ischemic cortex, which suppresses necroptotic signaling cascades and reduces proinflammatory cytokine release, representing a novel protein‐based therapy [304]. Targeting the RIP3/CaMKII/Pyk2 signaling axis, either via RIP3 deficiency or pharmacological inhibition of RIP3, alleviates necroptosis and apoptosis after IS by restoring mitochondrial membrane potential, reducing calcium influx, and suppressing ROS production [305]. Additionally, the FAAH inhibitor URB597 alleviates neuronal necroptosis by restoring autophagic flux in a PPARα‐dependent manner, reducing infarct volume and improving neurological function [306]. In TBI, emerging interventions also effectively inhibit necroptosis. The HSP70 activator TRC051384 suppresses HSP90α‐mediated phosphorylation of RIPK3 and MLKL, reducing trauma‐induced necroptotic cell death [307]. Perampanel, a selective AMPAR antagonist, decreases the expression and phosphorylation of RIPK1 and RIPK3 while activating the Akt/GSK3β pathway [308]. Omega‐3 polyunsaturated fatty acids act partially via the PPARγ/NF‐κB pathway to alleviate necroptosis and neuroinflammation, reduce cerebral edema, and improve neurological outcomes [183]. Finally, hydrogen‐rich saline reduces ROS levels and activates the heme oxygenase‐1 signaling pathway, downregulating RIP1/RIP3 expression and suppressing neuroinflammation after TBI [184].

Pyroptosis following IS can be alleviated by diverse novel therapeutic strategies. The selenium‐doped carbon dot nanozyme encapsulated in GSH‐conjugated liposomes scavenges ROS, upregulates GPX4, and suppresses the NLRP3/GSDMD signaling axis [309]. Transcranial direct current stimulation upregulates Netrin‐1 and activates the PPAR‐γ/NF‐κB pathway, thereby inhibiting NLRP3 inflammasome‐dependent pyroptosis [310]. Naomaili granules suppress endoplasmic reticulum stress and the RhoA/pyrin signaling pathway [311]. Artemisinin inhibits the ROS/TXNIP/NLRP3/Caspase‐1 pathway, reducing ROS production and mitochondrial membrane potential loss [312]. Ginsenoside Rg1 suppresses the CKLF1/CCR5 axis and downstream NF‐κB/NLRP3 inflammasome activation [313]. Elabela activates the APJ receptor, which suppresses ZBP1‐mediated NLRP3 activation and Drp1‐dependent mitochondrial fission [314]. Electroacupuncture activates SIRT5 to desuccinylate NEK7 at the K81 site, reducing its expression and inhibiting NLRP3 inflammasome activation [315]. An additional study confirmed that tDCS upregulates NTN‐1 via the PPAR‐γ/NF‐κB pathway to reduce postischemic pyroptosis [316]. Transcutaneous electrical acupoint stimulation inhibits the TLR4/MyD88/NF‐κB pathway, thereby reducing NLRP3 activation, GSDMD cleavage, and IL‐1β/IL‐18 release [317]. In TBI, emerging interventions also effectively suppress neuronal pyroptosis. HET0016, a specific inhibitor of 20‐HETE synthesis, alleviates pyroptosis in the immature brain by suppressing the p38 MAPK/NF‐κB pathway, reducing NLRP3 inflammasome activation, Caspase‐1 cleavage, and GSDMD‐mediated pore formation [318]. Tauroursodeoxycholic acid inhibits the STING/NF‐κB signaling axis, blocks IRF3/NF‐κB nuclear translocation, and suppresses NLRP3 inflammasome‐mediated pyroptotic cell death [319]. Metformin activates the AMPK pathway and restores Mfn1‐dependent mitochondrial dynamics, thereby suppressing NLRP3 inflammasome activation and reducing oxidative stress [320].

Ferroptosis following IS can be alleviated by diverse novel therapeutic strategies. Hydroxysafflor Yellow A inhibits NCOA4‐mediated ferritinophagy, reducing iron‐dependent lipid peroxidation [321]. Inhibition of SPP1 using the specific inhibitor Compound 11 or genetic knockdown suppresses the PI3K/AKT pathway and reduces ACSL4‐mediated lipid peroxidation [322]. Additionally, mangiferin activates the NRF2/ARE pathway, upregulating GPX4, SLC7A11, and FTL [323]. Furthermore, electroacupuncture preserves TFEB activation and lysosomal function, reducing ferritinophagy and lipid peroxidation in a TFEB‐dependent manner [324]. Ziziphora clinopodioides flavonoids enhance FUNDC1‐dependent mitophagy to clear pathological TDP‐43 aggregates [325]. Similarly, Eucommia ulmoides extract suppresses DDIT4 expression and activates the PI3K/Akt pathway [326]. Mechanistically, moracin N activates the Keap1/Nrf2 pathway, downregulating ACSL4 and promoting glutathione synthesis [327]. Likewise, pterostilbene activates the Wnt/β‐catenin pathway, reducing iron accumulation and lipid peroxidation [328]. Notably, intranasal recombinant human Stanniocalcin‐1 interacts with calreticulin to activate the CAMKK2/AMPK/GSK‐3β/Nrf2 pathway [329]. Finally, salidroside activates the PINK1/Parkin axis to enhance mitophagy, reducing ROS, malondialdehyde, and ferrous ion accumulation [330]. Ferroptosis after TBI is also effectively suppressed by emerging interventions. Neutralization of IL‐23 or blockade of CCL2 disrupts the CCL2–macrophage–IL‐23 axis and inhibits IL‐23 receptor‐mediated JAK2/STAT3 activation [331]. Additionally, herbacetin upregulates the HIF‐1α/SLC7A11/GPX4 axis, reducing lipid peroxidation and iron accumulation [332]. Moreover, paeoniflorin promotes P53 ubiquitination and inhibits its acetylation, derepressing SLC7A11 and activating the GPX4 pathway [333]. Furthermore, intermittent fasting upregulates GPX4 and HSPB1 while reducing TBI‐induced elevations of Nfe2l2, Slc7a11, Alox8, Steap3, and Nox2 [334]. Similarly, propofol modulates the eNOS/NO pathway, reducing iron deposition and neuroinflammation [335]. Mechanistically, activation of PPARγ by its specific agonist pioglitazone downregulates Cyclooxygenase‐2 expression [336]. In addition, AdipoRon, an adiponectin receptor agonist, activates APN–AdipoR1 signaling and promotes AMPK‐mediated phosphorylation of ACC1, suppressing polyunsaturated fatty acid biosynthesis [337]. Likewise, N‐Acetyl serotonin, a TrkB agonist, activates the TrkB/PI3K/Akt/Nrf2/Fth pathway [338]. Finally, electroacupuncture upregulates xCT expression and activates the System Xc^−^/GSH/GPX4 axis [339].

#### 4.3.2 Therapeutic Approaches Targeting Glutamate Excitotoxicity

Given the central role of glutamate excitotoxicity in driving secondary neuronal death after both IS and TBI, emerging therapeutic strategies have moved beyond direct NMDA receptor blockade toward more nuanced modulation of glutamate homeostasis, receptor trafficking, and downstream survival signaling. In IS, several novel compounds and biological approaches have demonstrated efficacy. The salidroside derivative SHPL‐49 attenuates excitotoxicity by promoting GLT‐1‐mediated glutamate reuptake while activating the NR2A–CaMKIIα–Akt/CREB survival axis, thereby extending the therapeutic window [340]. Similarly, the cryptotanshinone derivative DST‐3 preserves calcium homeostasis and mitochondrial function via the CREB‐Homer1 pathway [341]. Epigenetic modulation targeting Set7/9 disrupts the Cbx1–H3K9me2/3 repressive complex at the Sirt5 promoter, restoring Sirt5 expression and reducing glutaminase‐driven glutamate and ammonia accumulation [342]. Modulation of the ciRS‐7/miR‐7 axis alleviates aberrant glutamatergic signaling in excitatory pyramidal neurons [343]. Additionally, the interfering peptide TAT–M2PBM disrupts TRPM2–PKCγ interaction, attenuating extrasynaptic NMDAR‐mediated excitotoxicity without compromising normal PKCγ function [344]. The natural coumarin from Amburana cearensis reduces ROS production and downregulates the ERK/MAPK pathway, inhibiting Caspase‐3 activation under glutamate or OGD [345].

In TBI, analogous innovative interventions have emerged. THSG, a water‐soluble compound from Polygonum multiflorum, reduces L‐glutamate‐mediated DNA fragmentation, attenuates apoptosis, and promotes neurogenesis [346]. Targeting the CB1 receptor with AM281 reverses 2‐AG/CB1‐mediated suppression of GLT‐1 expression via the CREB pathway, thereby alleviating posttraumatic excitotoxicity and cognitive dysfunction [210]. Pharmacological inhibition of spermine oxidase with JNJ‐9350 restores astrocytic GLT‐1 membrane localization and glutamate clearance in the aged brain [128]. Bone marrow mesenchymal stem cell‐derived exosomes (BMSCs‐Exos) upregulate GLT‐1 expression by blocking p38 MAPK signaling, reducing neuronal apoptosis, lesion volume, and cognitive deficits [347]. Finally, the low‐molecular‐weight compound edonerpic maleate inhibits surface NR2B and GluR1 expression, reduces intracellular Ca^2^^+^ responses, and modulates CRMP2 and Arc signaling to improve long‐term neurological outcomes after TBI [348]. Collectively, these diverse pharmacological, epigenetic, and cell‐based strategies highlight that targeting glutamate excitotoxicity through pathway‐specific mechanisms holds substantial promise for limiting secondary injury and improving recovery in both IS and TBI.

#### 4.3.3 Therapeutic Approaches Targeting Oxidative Stress

Accumulating evidence has established oxidative stress as a central driver of secondary neuronal damage in both IS and TBI, prompting the development of diverse therapeutic strategies that target ROS production, antioxidant defense systems, and interconnected inflammatory and cell death pathways. In IS, several natural compounds and repurposed agents have demonstrated efficacy by modulating key oxidative stress‐related signaling axes. Glabridin, a flavonoid from licorice, activates the PI3K/Akt pathway to reduce MDA, increase SOD activity, and suppress inflammation and apoptosis [349]. ITLN‐1 overexpression or treatment with the small molecule AKR‐501 alleviates oxidative stress and mitochondrial dysfunction via mGluR7 binding and regulation of MAPK cascades [350]. Mangiferin activates the NRF2/ARE pathway, reducing ROS and MDA, restoring GSH and SOD, and inhibiting ferroptosis and neuroinflammation [323]. Cyanidin‐3‐glucoside directly targets NOX4 and modulates the Nrf2–Keap1–HO‐1 antioxidant axis, thereby reducing infarct volume and cerebral edema [351]. Silibinin protects through EGFR/ERK activation, downregulating Bax and cleaved Caspase‐3 while upregulating Bcl‐2 [294]. Cimifugin reduces MDA, restores SOD/GSH, and suppresses neuroinflammation and apoptosis [352]. 20(R)‐ginsenoside Rg3 inhibits the TLR4/MyD88/NF‐κB pathway to attenuate both neuroinflammation and oxidative stress [353]. Calcitriol reduces ROS, MDA, and NO, increases total antioxidant capacity, and blocks the ROS/TXNIP/NLRP3 inflammasome axis [354]. β‐Caryophyllene modulates p38MAPK/NF‐κB signaling, lowering MDA, elevating SOD, and suppressing TNF‐α/IL‐1β release [355].

In TBI, analogous antioxidant‐based interventions have emerged. Amlexanox, a broad‐spectrum PDE inhibitor, alleviates oxidative and ER stress by restoring lysosomal function via PKA activation and suppressing glial NF‐κB/STAT3 signaling [356]. L‐theanine reduces AMPA receptor‐mediated zinc accumulation, enhances glutathione synthesis, and mitigates neuroinflammation, thereby protecting hippocampal neurons [357]. Mirodenafil, a PDE5 inhibitor, restores cGMP levels, enhances nitric oxide signaling, and corrects zinc dysregulation [358]. Stigmasterol suppresses the STAT3/NF‐κB/LCN‐2 cascade, reducing neuroinflammation, brain edema, and cognitive deficits [301]. Dapagliflozin, an SGLT2 inhibitor, reduces ROS‐induced DNA damage and oxidative stress while restoring insulin pathway signaling and suppressing Aβ/p‐Tau pathology [359]. Hericium erinaceus mycelium and its derivative erinacine C activate the Nrf2‐mediated antioxidant pathway, upregulating catalase, glutathione reductase, thioredoxin reductase, SOD, and p‐CREB, thereby reducing microglial activation and neuronal death in mild TBI [360]. Collectively, these pharmacological, biological, and repurposed strategies highlight that targeting oxidative stress through pathway‐specific mechanisms, ranging from NRF2 activation and NOX4 inhibition to PDE modulation and metal ion homeostasis, holds substantial promise for limiting secondary injury and improving recovery in both IS and TBI (Tables 1 and 2).

**TABLE 1:** Therapeutic strategies targeting signaling pathways and major pathophysiological processes in preclinical IS models.

Therapeutic strategies	Targeting signaling pathway	Major targeting pathophysiology	Applications	Citations
Guanxinning	TLR‐4/NF‐κB/MMP‐9	BBB	HBMECs–OGD	[72]
Luteolin	MMP‐9	BBB	Mice‐MCAO; bEnd.3–OGD	[74]
Cepharanthine	VEGF/VEGFR2	BBB	Mice‐MCAO; bEnd.3–OGD	[121]
Danhong injection	Wnt/β‐Catenin	BBB	Rats‐MCAO	[239]
Trifluoperazine	MLCK/p‐MLC	BBB	Mice‐MCAO; bEnd.3–OGD	[240]
Rosmarinic acid	MMP‐2/MMP‐9	BBB	Mice‐MCAO	[241]
L‐Borneolum	Mfsd2a/Caveolin‐1	BBB	Rats‐MCAO; hCMEC–OGD	[245]
Recombinant human FGF17	Nrf2/HO‐1	BBB	Mice‐MCAO; bEnd.3–OGD	[247]
P23	GPX4	BBB	Mice‐MCAO; bEnd.3–OGD	[248]
Se‐(Methyl)‐selenocysteine	Akt/GSK3β	BBB	Mice‐MCAO; bEnd.3–OGD	[249]
Endothelial cell‐derived exosomes	PDGF–PDGFRβ	BBB	Mice‐MCAO	[252]
Edaravone dexborneol	PDGF–PDGFRβ	BBB	Rats‐MCAO	[253]
SKF‐86002	CX43/AQP4	BBB	Mice‐MCAO	[257]
Wuling san	TRPM2/NLRP3	BBB	Mice‐MCAO	[260]
Sulforaphane	Nrf2/MMP‐9	BBB	Mice‐MCAO	[361]
Empagliflozin	Wnt/β‐catenin	BBB	Mice‐MCAO; bEnd.3–OGD	[362]
Cinnamon	Calpain/VEGF	BBB	Rats‐MCAO	[363]
Nuciferine	JAK2/STAT3	BBB	Mice‐MCAO	[364]
Rosmarinic acid liposomes	ACSL4/LPCAT3/LOX	BBB	Mice‐MCAO	[365]
SEW2871	S1PR1/ERK	BBB	Rats‐UCAO/JPD	[366]
CAY10444	S1PR3/PI3K/Akt	BBB	Mice‐MCAO	[367]
Lifibrate	MLCK/p‐MLC	BBB	Mice‐MCAO; bEnd.3–OGD	[368]
1, 2,4‐Triazole	MMP‐9	BBB	Mice‐MCAO	[369]
Melatonin	PI3K/Akt/Nrf2	BBB	Rats‐MCAO	[370]
Axitinib	VEGF/VEGFR	BBB	Rats‐MCAO; bEnd.3–OGD	[371]
MDL28170	MMP‐2/MMP‐9	BBB	Rats‐MCAO	[372]
Melatonin	MMP‐9	BBB	Rats‐MCAO	[373]
Litebamine	MMP‐2/MMP‐9	BBB	Mice‐MCAO; bEnd.3–OGD	[374]
Netrin‐5	Wnt3a/β‐catenin	BBB	Mice‐MCAO; HBMEC–OGD	[375]
CP‐10	FRP1/NF‐κB	Neuroinflammation	Mice‐MCAO	[132]
KLF4	NF‐κB	Neuroinflammation	Mice‐MCAO; primary astrocyte–OGD	[154]
β‐Elemene	TLR4/NF‐κB	Neuroinflammation	Mice‐MCAO; BV2–LPS–IFN‐γ	[264]
Formononetin	TLR4/NF‐κB	Neuroinflammation	BV2–OGD	[265]
Glibenclamide	TLR4/NF‐κB	Neuroinflammation	Mice‐MCAO	[266]
Tongfu‐xingshen capsule	RhoA/ASC/GSDMD	Neuroinflammation	Rats‐MCAO; BV2–OGD	[269]
Edaravone dexborneol	CXCL13/CXCR5/NF‐κB	Neuroinflammation	Rats‐MCAO; primary microglia–OGD	[270]
ST909	PI3K/Akt	Neuroinflammation	Rats‐MCAO	[271]
Resveratrol	Epac‐1/Rap‐1	Neuroinflammation	Rats‐MCAO	[272]
Melatonin	cGAS/STING/NF‐κB	Neuroinflammation	Mice‐MCAO	[282]
Huang‐lian‐jie‐du decoction	LFA‐1/ICAM‐1	Neuroinflammation	Rats‐MCAO	[283]
Jolkinolide B	JAK2/STAT3	Neuroinflammation	Rats‐MCAO; HAPI–OGD	[376]
DCPIB	MAPK	Neuroinflammation	Mice‐MCAO	[377]
Semaglutide	NF‐κB	Neuroinflammation	Rats‐MCAO	[378]
CAY10444	S1PR3/ERK/p38MAPK	Neuroinflammation	Mice‐MCAO	[379]
Fingolimod	S1PR3/TLR2/4/NF‐κB	Neuroinflammation	Primary astrocyte–OGD	[380]
14, 15‐Epoxyeicosatrienoic acid	Wnt/β‐Catenin	Neuronal apoptosis	Mice‐MCAO; HT22–OGD	[290]
miR‐495‐3p	Wnt/β‐Catenin	Neuronal apoptosis	Rats‐MCAO; HT22–OGD	[291]
BMSCs‐derived exosomal Egr2	RNF8/DAPK1	Neuronal apoptosis	N2a–OGD	[292]
miR‐125a‐5p	TLR4/NF‐κB	Neuronal apoptosis	Rats‐MCAO; HT22–OGD	[293]
Silibinin	EGFR/ERK	Neuronal apoptosis	Mice‐MCAO	[294]
N‐acetylcysteine	NGF/Akt	Neuronal apoptosis	Rats‐MCAO	[295]
Sulbutiamine	MAPK	Neuronal apoptosis	Rats‐MCAO; SH‐SY5Y–OGD	[381]
Flavonoids	Keap1/Nrf2	Neuronal apoptosis	Mice‐MCAO; PC12–OGD	[382]
Q‐VD‐OPH	Nrf2/NQO1	Neuronal necroptosis	Rats‐MCAO	[303]
URB597	PPARα	Neuronal necroptosis	Mice‐MCAO	[306]
Selenium‐doped carbon dots nanozymes	GPX4/ROS/NLRP3/GSDMD	Neuronal pyroptosis	Mice‐MCAO	[309]
Transcranial direct current stimulation	PPAR‐γ/NF‐κB	Neuronal pyroptosis	Rats‐MCAO	[310]
Naomaili granules	RhoA/Pyrin	Neuronal pyroptosis	Rats‐MCAO	[311]
Ginsenoside Rg1	CKLF1/CCR5	Neuronal pyroptosis	Rats‐MCAO; PC12–OGD	[313]
Elabela	APJ/ZBP1	Neuronal pyroptosis	Mice‐MCAO; HT‐22–OGD	[314]
Electroacupuncture	SIRT5/NEK7	Neuronal pyroptosis	Mice‐MCAO	[315]
hUMSC‐derived exosomes	AMPK/NLRP3	Neuronal pyroptosis	Mice‐MCAO	[316]
Transcutaneous electrical acupoint stimulation	TLR‐4/MyD88/NF‐κB	Neuronal pyroptosis	Rats‐MCAO	[317]
T817MA	Sirt1	Neuronal pyroptosis	Rats‐MCAO	[383]
Resolvin E1	ChemR23	Neuronal pyroptosis	Mice‐MCAO; SH‐SY5Y–OGD	[384]
Compound 11	SPP1/PI3K/Akt	Neuronal ferroptosis	Mice‐MCAO; HT22–OGD	[322]
Mangiferin	Nrf2/ARE	Neuronal ferroptosis	Rats‐MCAO	[323]
Electroacupuncture	mTORC1/TFEB	Neuronal ferroptosis	Rats‐MCAO	[324]
Ziziphora clinopodioides flavonoids	FUNDC1/TDP‐43	Neuronal ferroptosis	Rats‐MCAO; PC12–OGD	[325]
Eucommia ulmoides extract	PI3K/Akt	Neuronal ferroptosis	Mice‐MCAO; SH‐SY5Y–OGD	[326]
Moracin N	Keap1/Nrf2	Neuronal ferroptosis	Mice‐MCAO	[327]
Pterostilbene	Wnt/β‐Catenin	Neuronal ferroptosis	Mice‐MCAO; HT22–OGD	[328]
Staniocalcin‐1	CAMKK2/AMPK/GSK‐3β/Nrf2	Neuronal ferroptosis	Mice‐MCAO	[329]
Salidroside	PINK1/Parkin	Neuronal ferroptosis	Rats‐MCAO; SH‐SY5Y–OGD	[330]
NEDD8	Nrf2/FTH1	Neuronal ferroptosis	HT22–OGD	[385]
Tetrahydrocurcumin	Keap1/Nrf2	Neuronal ferroptosis	Mice‐MCAO	[386]
Rosmarinic acid	Keap1/Nrf2	Neuronal ferroptosis	Rats‐MCAO	[387]
Zha‐chong shi‐san‐wei pill	HIF‐1α/HO‐1	Neuronal ferroptosis	Rats‐MCAO; PC12–OGD	[388]
Salidroside derivative SHPL‐49	NR2A/CAMK IIα/Akt	Glutamate excitotoxicity	Rats‐MCAO; primary neuronal cell–OGD	[340]
DST‐3	CREB/Homer 1	Glutamate excitotoxicity	Rats‐MCAO	[341]
Coumarin	MAPK	Glutamate excitotoxicity	PC12–OGD	[345]
Silibinin	EGFR/ERK	Oxidative stress	Mice‐MCAO	[294]
Mangiferin	Nrf2/ARE	Oxidative stress	Rats‐MCAO	[323]
Glabridin	PI3K/Akt	Oxidative stress	Mice‐MCAO; HT22–OGD	[349]
AKR‐501	ITLN‐1/mGluR7/MAPK	Oxidative stress	Mice‐MCAO	[350]
Cyanidin‐3‐glucoside	Nrf2–Keap1–HO‐1	Oxidative stress	Mice‐MCAO; HT22–OGD	[351]
Cimifugin	iNOS/COX‐2	Oxidative stress	Rats‐MCAO	[352]
20(R)‐ginsenoside Rg3	TLR4/MyD88/NF‐κB	Oxidative stress	Rats‐MCAO	[353]
Calcitriol	ROS/TXNIP/NLRP3	Oxidative stress	Rats‐MCAO	[354]
β‐Caryophyllene	p38MAPK/NF‐κB	Oxidative stress	Rats‐MCAO	[355]
CAY10444	S1PR3/nNOS/NO	Oxidative stress	Mice‐MCAO	[389]

**TABLE 2:** Therapeutic strategies targeting signaling pathways and major pathophysiological processes in preclinical TBI models.

Therapeutic strategies	Targeting signaling pathway	Major targeting pathophysiology	Applications	Citations
Tauroursodeoxycholic acid	IRE1/CXCL10	BBB	Mice‐CCI	[64]
Thyroid hormone T4	MMP‐9	BBB	Mice‐CCI	[71]
Cystamine	IL‐17/MMP‐9	BBB	Mice‐CCI; bEnd.3–OGD	[75]
CYM‐5442	S1PR1/Mfsd2a/Caveolin‐1	BBB	Mice‐CCI	[77]
Atorvastatin‐loaded PEGylated liposomes	Caspase‐3	BBB	Mice‐CCI	[92]
Ferrostatin‐1	GPX4/COX‐2	BBB	Mice‐CCI; bEnd.3‐stretch injury	[103]
miR‐211‐5p	AQP4	BBB	Rats‐CCI	[125]
JTE‐013	S1PR2/PI3K/Akt	BBB	Mice‐CCI	[242]
Fucoxanthin	MMP‐9	BBB	Mice‐CCI; bEnd.3‐stretch injury	[243]
Fluvoxamine	AQP4	BBB	Mice‐CCI	[261]
Minocycline	AQP4	BBB	Mice‐CCI	[262]
Astragaloside	Akt/FoxO3a/AQP4	BBB	Mice‐CCI	[263]
Recombinant human FGF17	Akt/GSK‐3β	BBB	Mice‐CCI; HBMEC–TNF‐α	[390]
Carvacrol	JMJD3‐SURI/TRPM4	BBB	Mice‐CCI	[391]
BMS309403	JNK/c‐Jun/MMP‐12	BBB	Mice‐CCI	[392]
Bevacizumab	VEGF/MMP‐9	BBB	Rats‐CCI	[393]
JTE‐013	S1PR2/JNK/c‐Jun/MMP‐9	BBB	Mice‐CCI	[394]
Fingolimod	S1PR1/ERK	BBB	Mice‐weight drop model	[395]
Recombinant human FGF20	Wnt/β‐catenin	BBB	Mice‐CCI	[396]
VCE‐004.8	PP2A/B55α/PHD2/HIF	BBB	Mice‐CCI	[397]
17‐DA	MMP‐2/MMP‐9	BBB	Mice‐CCI	[398]
Doxycycline	MMP‐9	BBB	Mice‐CCI	[399]
miR‐124‐3p	mTOR	BBB	bEnd.3‐scratch injury	[400]
G1	GPER1/Mcp‐1/MIP‐2	Neuroinflammation	Mice‐CCI	[144]
Esketamine	METTL5/c‐Myc/PD‐L1	Neuroinflammation	Mice‐CCI	[163]
GSK2795039	NOX2/ROS/NLRP3	Neuroinflammation	Mice‐CCI; IMG‐LPS	[170]
β‐Asarone	Fas/FasL	Neuroinflammation	Mice‐CCI	[274]
VitaminD	TLR4/MyD88/NF‐κB	Neuroinflammation	Rats‐CCI	[275]
Electromagnetic field exposure	TNF‐α	Neuroinflammation	HMC3–TNF‐α	[276]
C34	TLR4	Neuroinflammation	Mice‐CCI	[278]
Remazolam	CX43	Neuroinflammation	BNCC337745–OGD	[279]
Rovelizumab	ITGB2/ICAM‐1	Neuroinflammation	Mice‐FPI	[286]
Esketamine	STAT3	Neuroinflammation	Mice‐CCI	[287]
Nab‐CXCL5	CXCL5/CXCR2	Neuroinflammation	Mice‐CCI	[288]
VCE‐005.1	AMPK/HIF‐1α	Neuroinflammation	Mice‐CCI	[401]
MLN4924	NEDD8/TRIM56/STING	Neuronal apoptosis	Mice‐CCI	[8]
RVG29‐conjugated nanoparticles	FNDC5/Nrf2/SIRT3	Neuronal apoptosis	Mice‐CCI	[296]
GC/I/E hydrogel	G3‐FFAP	Neuronal apoptosis	Mice‐CCI; HT22‐H_2_O_2_	[297]
Zileuton	ERK/NF‐κB/Akt	Neuronal apoptosis	Mice‐CCL	[298]
Nle4‐D‐Phe7‐α‐melanocyte	MC1R/Merlin	Neuronal apoptosis	Rats‐CCI	[299]
ZLN005	PGC‐1α/OXPHOS	Neuronal apoptosis	Mice‐CCI; HT22‐TNF‐α	[300]
Stigmasterol	STAT3/NF‐κB/LCN‐2	Neuronal apoptosis	Rats‐CCI; HT22‐H_2_O_2_	[301]
Resveratrol	ERS	Neuronal apoptosis	Mice‐CCI	[302]
Omega‐3 polyunsaturated fatty acids	PPARγ/NF‐κB	Neuronal necroptosis	Mice‐CCI	[183]
Hydrogen‐rich saline	ROS/HO‐1	Neuronal necroptosis	Mice‐weight drop model	[184]
TRC051384	RIPK3/MLKL	Neuronal necroptosis	Cortical primary neuron‐glutamate	[307]
Perampanel	Akt/GSK‐3β	Neuronal necroptosis	Cortical primary neuron‐scratch injury	[308]
HET0016	p38MAPK	Neuronal pyroptosis	Rats‐CCI	[318]
Tauroursodeoxycholic acid	STING/NF‐κB	Neuronal pyroptosis	Mice‐CCI	[319]
Metformin	AMPK	Neuronal pyroptosis	HT22‐scratch injury	[320]
10‐hydroxy‐2‐decenoic acid	ATP7A	Neuronal pyroptosis	Mice‐CCI	[402]
Irisin	AMPK/MerTK	Neuronal pyroptosis	Mice‐CCI	[403]
Anti‐IL‐23 antibody	JAK2/STAT3	Neuronal ferroptosis	Mice‐CCI	[331]
Herbacetin	HIF‐1α/SLC7A11/GPX4	Neuronal ferroptosis	Mice‐CCI	[332]
Paeoniflorin	SLC7A11/GPX4	Neuronal ferroptosis	Mice‐CCI; cortical primary neuron‐erastin	[333]
Intermittent fasting	GPX4/Hspb1	Neuronal ferroptosis	Mice‐CCI	[334]
Propofol	eNOS/NO	Neuronal ferroptosis	Mice‐weight drop model	[335]
Pioglitazone	PPARγ/COX‐2	Neuronal ferroptosis	Mice‐weight drop model; HT22‐RAS‐selective lethal 3	[336]
AdipoRon	AMPK/ACC1	Neuronal ferroptosis	Mice‐CCI; cortical primary neuron‐erastin	[337]
N‐acetyl serotonin	PI3K/Akt/Arf2/Ferritin H	Neuronal ferroptosis	Mice‐CCI	[338]
Electroacupuncture	GSH/GPX4	Neuronal ferroptosis	Rats‐weight drop model	[339]
NL‐1	mitoNEET/DHODH	Neuronal ferroptosis	Mice‐CCI	[404]
Butylphthalide	AHR/CYP1B1	Neuronal ferroptosis	Mice‐CCI	[405]
JNJ‐9350	SMOX	Glutamate excitotoxicity	Mice‐CCI	[128]
AM281	CB1/CREB	Glutamate excitotoxicity	Mice‐CCI	[210]
Bone marrow stromal cells‐derived exosomes	p38MAPK/GLT‐1	Glutamate excitotoxicity	Rats‐CCI; cortical primary neuron‐glutamate	[347]
Edonerpic maleate	CRMP2/Arc	Glutamate excitotoxicity	Rats‐CCI; cortical primary neuron‐glutamate and scratch injury	[348]
Stigmasterol	STAT3/NF‐κB/LCN‐2	Oxidative stress	Rats‐weight drop model; HT22‐H_2_O_2_	[301]
Amlexanox	NF‐κB/STAT3	Oxidative stress	Rats‐CCI; cortical primary neuron‐scratch injury	[356]
L‐theanine	AMPA	Oxidative stress	Rats‐CCI; cortical primary neuron‐scratch injury	[357]
Mirodenafil	Nrf2/HO‐1	Oxidative stress	Rats‐CCI; cortical primary neuron‐zinc chloride	[358]
Dapagliflozin	SGLT2/ERK	Oxidative stress	SH‐SY5Y‐stretch injury	[359]
Novel erinacines from *Hericium erinaceus* mycelium	Nrf2	Oxidative stress	Rats‐weight drop model	[360]

Despite the substantial preclinical evidence supporting the efficacy of numerous antioxidant and neuroprotective strategies in animal models of IS and TBI, translation to clinical practice has been overwhelmingly disappointing limited. This translational gap stems from multiple interconnected factors. The heterogeneity of TBI, encompassing variable injury mechanisms, severity, and anatomical locations, renders uniform therapeutic approaches inherently challenging, a complexity that is rarely captured in preclinical models. The therapeutic time window for antioxidant interventions is exceptionally narrow, with most agents showing efficacy only when administered within minutes to hours of injury, a timeframe that is difficult to achieve in clinical practice. Additionally, the BBB, which may be compromised to varying degrees after injury, poses a significant pharmacokinetic obstacle, limiting the bioavailability of many promising compounds. The failure to stratify patients based on baseline oxidative burden or inflammatory status may have diluted potential benefits in heterogeneous trial populations. The assumption that nonspecific scavenging of ROS is therapeutically beneficial may be fundamentally flawed, as ROS serve essential physiological signaling functions, and their indiscriminate elimination may disrupt adaptive redox responses. Emerging strategies that may improve translational prospects include targeted inhibition of specific ROS‐generating enzymes, activation of endogenous antioxidant pathways, mitochondria‐targeted antioxidants, and combination therapies that address multiple nodes of the secondary injury cascade simultaneously. Collectively, these considerations underscore the urgent need for well‐designed clinical trials to evaluate the safety and efficacy of mechanism‐based interventions targeting the core pathophysiological processes of secondary brain injury, as discussed in the following section.

## 5 Emerging Clinical Trials

In parallel with preclinical developments, a growing number of clinical trials have been initiated to evaluate mechanism‐based interventions targeting the core pathophysiological processes of secondary brain injury, including BBB disruption, neuroinflammation, neuronal apoptosis, excitotoxicity, and oxidative stress, in both IS and TBI.

### 5.1 Clinical Trials Targeting Secondary Injury Pathways in IS

Several completed or ongoing trials have tested agents that modulate distinct pathological cascades after IS. For BBB protection, the GALLOP trial (NCT05920889) investigated semaglutide within 12 h of stroke onset, while the ANGONG trial (NCT04475328) assessed the Angong Niuhuang Pill given within 36 h. Noninvasive vagus nerve stimulation (NOVIS, NCT04050501; NUVISTA2, NCT07404852) has also been evaluated for its ability to preserve BBB integrity and modulate neuroinflammation.

Minocycline, a pleiotropic anti‐inflammatory and anti‐apoptotic agent, has been extensively studied in IS. The MIST‐A trial (NCT05487417) is recruiting patients to receive oral minocycline within 24 h, primarily targeting BBB stabilization and neuroinflammation. The completed EMPHASIS trial (NCT05836740) examined oral minocycline within 72 h, and the ATTRACTION‐MINOA (NCT07594314) and MIST‐B (NCT05512910) trials are further evaluating minocycline in patients with large‐vessel occlusion stroke, focusing on neuroinflammation and oxidative stress pathways.

Other notable trials targeting neuroinflammation include the IRIS‐3 study (NCT07509645) testing tocilizumab within 24 h, and the ExoCURE trial (NCT06138210) using GD‐iExo‐003 for patients with symptom onset of 1–7 days. For neuronal apoptosis, the IAT‐GIANT trial (NCT07519044) is investigating monosialotetrahexosylganglioside within 24 h, while the SICAS study (NCT06331494) evaluates butylphthalide within 7 days. Excitotoxicity is addressed by the xenon inhalation trial (NCT05335109) started within 8 h, and oxidative stress by the cytoflavin trial (NCT05935787) and the BLESS study (NCT07230587) using butylphthalide within 2 weeks. A comprehensive list of all IS trials, including their phases, sample sizes, doses, routes, and targeted pathological processes, is summarized below (Table 3).

**TABLE 3:** Clinical trials targeting pathophysiological processes in IS.

Trial number	Trial name	Current status	Trial time frame	Phase	Sponsor	Number of participants	Therapeutic strategies	Dose	Route	Time from stroke occur	Pathological process
NCT05920889	Glucagon‐like peptide 1 receptor agonist in acute large vessel occlusion stroke treated by reperfusion therapies (GALLOP)	Completed	2023.08.01–2024.10.25	2	University	140	Semaglutide	0.5 mg	SC	<12 h	BBB
NCT05487417	Effects of minocycline on patients with ischemic stroke undergoing intravenous thrombectomy (MIST‐A)	Recruiting	2022.11.01–2025.06.10	4	Hospital	180	Minocycline	200 mg	PO	≤24 h	BBB
NCT04475328	Efficacy and safety of AngongNiuhuang pill in patients with acute ischemic stroke (ANGONG TRIAL)	Completed	2020.10.01–2022.07.30	3	Hospital	120	AngongNiuhuang pill	1 pill	PO	≤36 h	BBB
NCT06737549	Effect of specific mode electroacupuncture stimulation combined with NGF on dysphagia after ischaemic stroke: a pilot randomized controlled trial	Recruiting	2024.12–2027.12	4	Hospital	144	Nerve growth factor injection	20 µg	IM	15–180 d	BBB
NCT06858436	SGLT2 inhibitor utilization re‐perfusion therapy (SUPER)	Not yet recruiting	2026.04.13–2027.10.31	4	Hospital	150	Canagliflozin	100 mg	PO	Not reported	BBB
NCT04050501	Non‐invasive vagus nerve stimulation in acute ischemic stroke (NOVIS)	Completed	2019.10.09–2025.05.05	Not applicable	University	150	Noninvasive vagus nerve stimulator	2 min	Transcutaneous	<12 h	BBB
NCT06777680	Palmitoylethanolamide and luteolin in patients with acute ischemic stroke	Not yet recruiting	2025.01–2026.12	Not applicable	Hospital	60	Coultramicronized palmitoylethanolamide + luteolin oral suspension	770 mg	PO	Not reported	Neuroinflammation
NCT05836740	Efficacy and safety of minocycline in patients with moderate to severe acute ischemic stroke (EMPHASIS)	Completed	2023.05.19–2024.08.15	3	Hospital	1724	Minocycline	200 mg, 100 mg	PO	<72 h	Neuroinflammation
NCT07594314	Minocycline after successful endovascular thrombectomy recanalization in acute anterior circulation large vessel occlusion (ATTRACTION‐MINOA)	Not yet recruiting	2026.05–2028.12	2, 3	Hospital	860	Minocycline	200 mg, 100 mg	PO	<24 h	Neuroinflammation
NCT07121569	Oral DLBS1033 as adjunctive therapy in acute ischemic stroke: impact on inflammatory biomarkers and outcomes	Completed	2024.07.06–2025.03.13	4	University	52	DLBS1033	490 mg	PO	24 h–7 d	Neuroinflammation
NCT07404852	Non‐invasive vagus nerve stimulation to reduce inflammation and brain injury blood biomarkers following an acute ischemic stroke (NUVISTA2)	Recruiting	2026.02.23–2028.02.29	Not applicable	University	65	Transcutaneous auricular vagal nerve stimulation	20 min	Transcutaneous	Not reported	Neuroinflammation
NCT05390580	Neuromodulation using vagus nerve stimulation following ischemic stroke as therapeutic adjunct (NUVISTA)	Completed	2022.09.26–2024.08.23	Not applicable	University	40	Transcutaneous auricular vagal nerve stimulation	20 min	Transcutaneous	Not reported	Neuroinflammation
NCT06129175	UCMSCs as treatment for patients with acute ischemic stroke (stroke neuroncell‐EX)	Recruiting	2023.12.19–2026.12.31	2, 3	Industry	80	Neuroncell‐EX	2 million UCMSCs/kg	IV	<4 week	Neuroinflammation
NCT06138210	The effect of GD‐iExo‐003 in acute ischemic stroke (ExoCURE)	Recruiting	2024.06.15–2026.03.30	1, 2	Hospital	29	GD‐iExo‐003	3 × 10^11^ particles	IV	1–7 d	Neuroinflammation
NCT04952064	An exploratory study of ganglioside GM1 in acute ischemic stroke	Completed	2021.07.28–2022.07.18	4	Hospital	300	Ganglioside GM1	200 mg	IV	<24 h	Neuroinflammation
NCT07509645	Efficacy and safety of interleukin‐6 receptor inhibitor combined with endovascular treatment in patients with acute posterior circulation large vessel occlusion stroke (IRIS‐3)	Recruiting	2026.04.22–2027.04.20	3	University	348	Tocilizumab	240 mg	IV	<24 h	Neuroinflammation
NCT05512910	Minocycline for acute ischemic stroke undergoing endovascular treatment due to basilar artery occlusion (MIST‐B) (MIST‐B)	Active, not recruiting	2023.03.04–2025.06.04	4	Hospital	90	Minocycline	200 mg, 100 mg	IV	<24 h	Neuroinflammation
NCT07301372	Evaluation study of early administration of evolocumab after thrombolysis in patients with atherosclerotic acute ischemic stroke (EPOCH‐LYSIS)	Completed	2025.10.30–2026.04.29	4	University	132	Evolocumab	420 mg	SC	<4.5 h	Neuroinflammation
NCT06540417	Study of the effects of the non‐invasive trigeminal nerve stimulation on ischemic stroke patients	Completed	2024.07.25–2024.12.20	Not applicable	University	60	External trigeminal nerve stimulation	>8 h	Transcutaneous	<72 h	Neuroinflammation
NCT06920706	Efficacy of remote ischemic conditioning in preventing post‐stroke depression	Not yet recruiting	2025.04.10–2026.12.31	Not applicable	Hospital	240	Remote ischemic conditioning	5 min	RIC	≤48 h	Neuroinflammation
NCT07105917	SLGT2i for neuroprotection in patients with acute ischemic stroke	Not yet recruiting	2025.08.01–2026.11.01	3	Hospital	1050	Dapagliflozin or empagliflozin	10 mg	Not reported	≤3 d	Neuronal apoptosis
NCT07519044	Safety and efficacy of adjunctive GM1 to mechanical thrombectomy for acute anterior circulation large vessel occlusion (IAT‐GIANT)	Not yet recruiting	2026.04.30–2028.08.31	4	Hospital	868	Monosialotetrahexosylganglioside	200 mg	IV	<24 h	Neuronal apoptosis
NCT07205328	A clinical trial of MT200605 for the treatment of acute ischemic stroke	Recruiting	2025.10–2026.06	2	Industry	360	MT200605	10 mg	IV	<24 h	Neuronal apoptosis
NCT06331494	Efficacy of butylphthalide on symptomatic atherosclerotic stenosis in middle cerebral artery (SICAS)	Not yet recruiting	2024.03.15–2026.08.31	Not applicable	Hospital	140	Butylphthalide	100 mL, 0.2 g	IV, PO	<7 d	Neuronal apoptosis
NCT05335109	Neuroprotective effects of xenon treatment in patients with cerebral infarction	Completed	2022.01.01–2024.01.15	3	Institute	40	Xenon	30% concentration	Inhalation	<8 h	Glutamate excitotoxicity
NCT05935787	Cytoflavin in the complex rehabilitation of stroke patients	Completed	2023.06.30–2024.12.07	3	Industry	240	Cytoflavin	20 mL, 2 tabs	IV, PO	14–90 d	Oxidative stress
NCT07230587	Butylphthalide for long‐term efficacy in minor stroke study (BLESS)	Not yet recruiting	2025.12.01–2028.10.01	3	Hospital	1200	Butylphthalide	200 mg	PO	<2 week	Oxidative stress
NCT07594301	Minocycline after successful endovascular thrombectomy recanalization in posterior circulation arterial occlusion (ATTRACTION‐MINOP)	Not yet recruiting	2026.05–2028.12	2, 3	Hospital	234	Minocycline	200 mg, 100 mg	PO	≤24 h	Oxidative stress

*Data sources*: ClinicalTrials.gov.

### 5.2 Clinical Trials Targeting Secondary Injury Pathways in TBI

Clinical trials in TBI have similarly focused on mitigating secondary injury. For BBB preservation, the MATRIx trial (NCT06163833) is administering mesenchymal stromal cells to patients with severe TBI. Memantine, an NMDA receptor antagonist, is being evaluated in a Phase 2/3 trial (NCT06914310) for mild‐to‐moderate TBI, targeting both BBB function and neuronal apoptosis. Baricitinib (NCT06065046) is under investigation for moderate‐to‐severe TBI, with a focus on BBB protection.

Neuroinflammation is the most frequently targeted process in TBI trials. The VIMOT trial (NCT07269457) is testing vitamin D3 in mild‐to‐moderate TBI. Mesenchymal stromal cells derived from Wharton's jelly (TRAUMACELL, NCT06146062) and autologous adipose‐derived MSCs (NCT05951777) are being evaluated for immunomodulation. Noninvasive vagal nerve stimulation is examined in the VNS mTBI trial (NCT04437498) for mild TBI and the MINT trial (NCT07510074) for moderate‐to‐severe TBI. The Jianmin Daopeng Angong Niuhuang Wan (NCT07074938) is also being studied for postoperative recovery in moderate‐to‐severe TBI. Finally, the completed memantine hydrochloride trial (NCT06337994) addresses neuronal apoptosis in mild‐to‐moderate TBI. Detailed information on all TBI trials is available (Table 4).

**TABLE 4:** Clinical trials targeting pathophysiological processes in TBI.

Trial number	Trial name	Current status	Trial time frame	Phase	Sponsor	Number of participants	Therapeutic strategies	Dose	Route	TBI severity	Pathological process
NCT06163833	Mesenchymal stromal cells for traumatic brain injury (MATRIx)	Recruiting	2023.09.19–2026.12	2	Institute	78	Mesenchymal stromal cell low dosage	80 × 10^6^ cells, 160 × 10^6^ cells	IV	Severe	BBB
NCT06914310	Memantine as an additive therapy in patients with traumatic brain injury	Not yet recruiting	2025.03.25–2025.11.30	2, 3	University	80	Memantine	30 mg, 10 mg	PO	Mild to moderate	BBB
NCT06065046	Baricitinib for moderate and severe traumatic intracerebral hemorrhage/contusions	Recruiting	2023.11.24–2026.12.31	2	Hospital	100	Baricitinib	4 mg	PO	Moderate to severe	BBB
NCT07269457	Vitamin D3 for moderate to mild traumatic brain injury: a randomized trial on inflammation and recovery (VIMOT) (VIMOT)	Recruiting	2026.03.02–2028.12.30	2	University	240	Vitamin D3	2000 IU	PO	Mild to moderate	Neuroinflammation
NCT06146062	Effects of intravascular administration of mesenchymal stromal cells derived from Wharton's Jelly of the umbilical cord on systemic immunomodulation and neuroinflammation after traumatic brain injury (TRAUMACELL)	Recruiting	2024.06.25–2028.03.31	2	Hospital	68	Mesenchymal stromal cells (MSC)	2 × 10^6^/kg	IV	Severe	Neuroinflammation
NCT05951777	Autologous adipose‐derived mesenchymal stem cells for chronic traumatic brain injury	Recruiting	2024.04.16–2026.12	2	Industry	51	Autologous HB‐adMSCs	2 × 10^8^ cells	IV	Not reported	Neuroinflammation
NCT04437498	Vagal nerve stimulation in mTBI (VNS mTBI)	Recruiting	2021.02.25–2026.07.09	Not applicable	Institute	100	Noninvasive vagal nerve stimulation	Not reported	Transcutaneous	Mild	Neuroinflammation
NCT07074938	An Gong Niuhuang Wan promotes the postoperative neurological function recovery of patients with traumatic brain injury	Recruiting	2025.07.10–2028.07.31	Not applicable	Hospital	60	Jianmin Daopeng Angong Niuhuang Wan	1 pill	PO	Moderate to severe	Neuroinflammation
NCT07510074	Modulating inflammation in neuro‐trauma (MINT)	Not yet recruiting	2026.05–2027.03	Not applicable	University	16	Nurosym taVNS	Not reported	Transcutaneous	Moderate to severe	Neuroinflammation
NCT06337994	Memantine hydrochloride for treatment of cognitive dysfunction due to traumatic brain injury	Completed	2024.01.01–2024.08.01	3	University	60	Memantine hydrochloride	5 mg, 10 mg	PO	Mild to moderate	Neuronal apoptosis

*Data sources*: ClinicalTrials.gov.

Collectively, these ongoing and completed clinical trials underscore a paradigm shift from empiric neuroprotection to mechanism‐driven interventions that target specific secondary injury pathways. The diversity of agents and the range of pathological processes being addressed provide a rich pipeline for future therapeutic development in both IS and TBI.

## 6 Future Perspectives

Despite significant advances in understanding the pathophysiology of secondary brain injury after IS and TBI, effective mechanism‐specific therapies that directly target the secondary injury cascades remain limited, as reperfusion therapy is standard for eligible IS patients and TBI management is still largely supportive or surgical. No single neuroprotective agent has demonstrated consistent efficacy in clinical translation. This shortcoming arises from the highly interconnected nature of secondary injury cascades, where individual pathways are not isolated but rather engage in extensive crosstalk.

To overcome this challenge, future therapeutic strategies should prioritize the development of small molecule inhibitors that simultaneously target multiple converging pathways, including those regulating BBB disruption, neuroinflammation, and programmed cell death. In parallel, biomarker‐driven patient stratification will be essential to identify individuals most likely to benefit from specific mechanism‐based interventions, thereby moving beyond a uniform approach [406]. Novel delivery platforms, including extracellular vesicles engineered with targeting moieties and nanomaterial‐based carriers, offer the potential to enhance central nervous system bioavailability while reducing systemic off target effects [284, 407, 408, 409, 410, 411, 412]. Moreover, innovative routes of administration that circumvent the BBB, including intranasal delivery or direct transcranial administration, could provide more efficient access to injured brain parenchyma [413, 414, 415]. An emerging frontier lies in harnessing the gut–brain axis as an important strategy to regulate neurological function through the digestive system [416, 417, 418]. This approach serves as a valuable adjunctive therapy for effective management of neurological function after IS and TBI. In parallel, peripheral neuromodulation techniques, including vagus nerve stimulation and brain stimulation, have shown potential to improve secondary brain injury processes [419, 420, 421, 422] (Figure 4).

**FIGURE 4:**
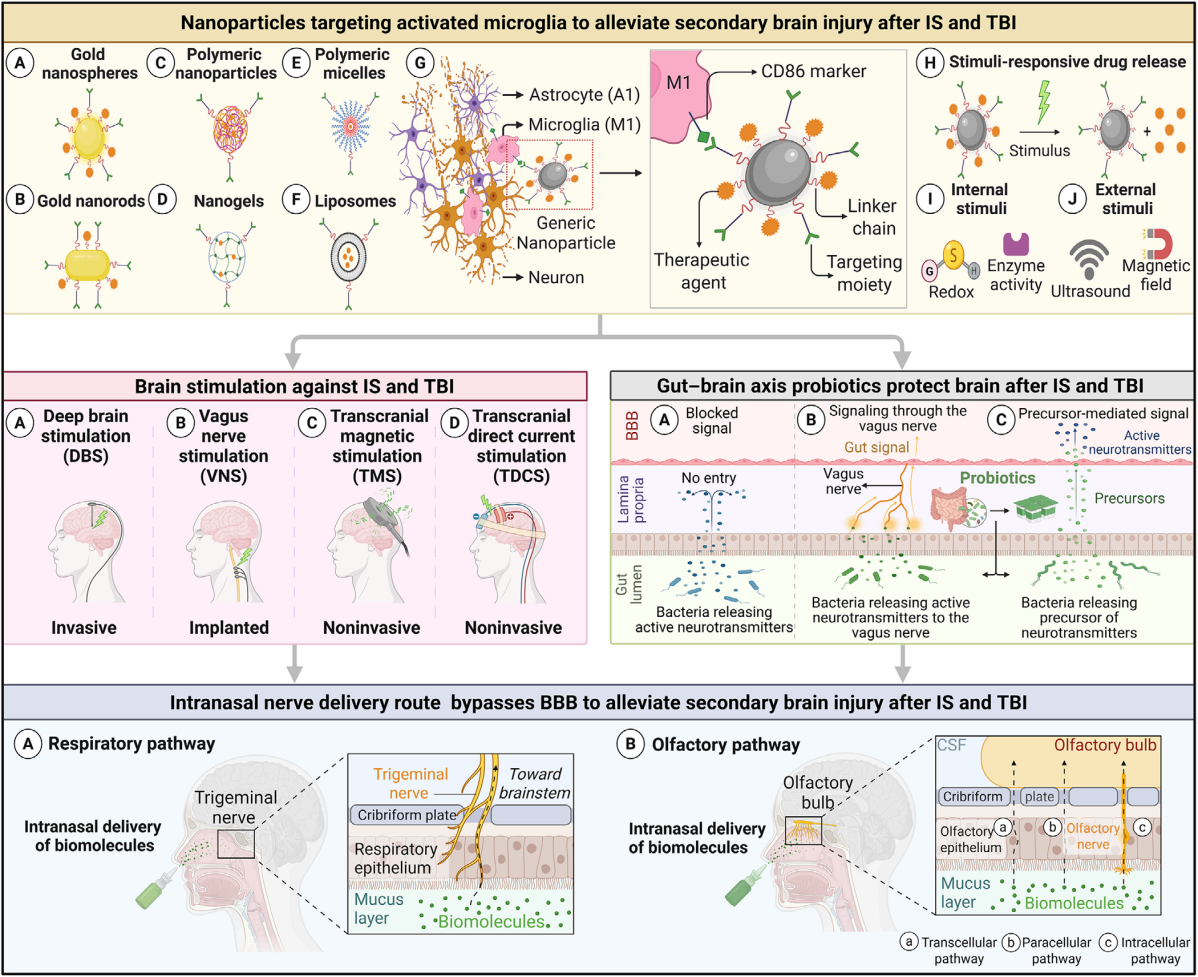
Emerging therapeutic strategies for secondary brain injury after IS and TBI. Future directions include nanomaterial‐based drug delivery to alleviate neuroinflammation, brain stimulation techniques, probiotic modulation of the gut–brain axis, and intranasal drug delivery that bypasses the BBB via the olfactory or trigeminal nerve routes. These innovative approaches hold promise for more effective and targeted treatment of IS and TBI.

Further validation of these interventions in clinical trials is warranted to confirm their therapeutic benefits. Collectively, these integrated approaches, ranging from multitarget pharmacology and advanced drug delivery to patient stratification and brain–gut nerve crosstalk, hold promise for achieving effective, multimodal, and personalized treatment of secondary brain injury after both IS and TBI.

## 7 Conclusion

Substantial advances have been made in understanding the pathophysiology of secondary brain injury after IS and TBI, with extensive preclinical evidence elucidating the interconnected cascades of BBB disruption, neuroinflammation, excitotoxicity, oxidative stress, and programmed cell death. However, clinical translation remains limited. Current therapeutic options for IS and TBI remain limited to reperfusion therapy in eligible IS patients and supportive or surgical management in TBI, and no single neuroprotective agent has demonstrated consistent efficacy in clinical trials. This translational gap arises from the highly interconnected nature of secondary injury cascades, where individual pathways engage in extensive crosstalk and positive feedback loops rather than operating in isolation.

Our comparative analysis reveals three principal insights. First, despite their different primary insults, IS and TBI converge on shared pathophysiological processes. Second, these pathways are highly dynamic and interconnected, forming self‐amplifying cascades that extend injury beyond the initial lesion. This explains why single‐target therapies have largely failed. Third, the spatial and temporal heterogeneity of these processes underscores the need for precisely timed and region‐specific interventions.

Several interconnected challenges impede clinical translation. The complex, multilevel nature of secondary injury demands therapeutic strategies capable of simultaneous pathway modulation, yet most current approaches remain single‐target oriented. The absence of validated biomarkers prevents reliable patient stratification, making it difficult to identify individuals most likely to benefit from specific mechanism‐based interventions. Additionally, the restricted bioavailability of therapeutic agents due to limited BBB penetration, combined with the narrow therapeutic time window, severely constrains the clinical applicability of many promising candidates. These challenges collectively highlight the urgent need for more integrated and precisely targeted therapeutic frameworks.

Addressing these challenges will require a paradigm shift toward multimodal, mechanism‐based therapeutic strategies. Key priorities include biomarker‐driven patient stratification, advanced drug delivery systems that bypass or cross the BBB, and combination regimens that target multiple interconnected pathways simultaneously. Emerging approaches such as gut–brain axis modulation and peripheral neuromodulation also hold promise as adjunctive strategies. Further validation of these interventions in well‐designed clinical trials is warranted to confirm their therapeutic benefits. Collectively, these integrated approaches hold promise for achieving effective, multimodal, and personalized treatment of secondary brain injury in both IS and TBI.

## Author Contributions

Study conception and design: Mengzhao Feng and Qi Qin. Literature collection: Fang Wang and Kaiyuan Zhang. Summarize the findings: Mengzhao Feng and Qi Qin. Draft manuscript preparation: Mengzhao Feng and Qi Qin. Critical revision of the article: Fuyou Guo, Min Yu, Junlei Chang, and Zhihua Li. All authors reviewed the results and approved the final version of the manuscript.

## Funding

This work was funded with these programs: National Natural Science Foundation of China (32471043), Leading Project of Henan Province Young Medical Research (LJRC2023010), National Key R&D Program of China (2023YFE0202200), Guangdong Science and Technology Program (2025B1212150002), Shenzhen Medical Research Fund (D2403002), and National Natural Science Foundation of China (82503692).

## Ethics Statement

This manuscript does not involve any new studies of human or animal subjects performed by any of the authors.

## Conflicts of Interest

The authors declare no conflicts of interest.

## Data Availability

No datasets were generated or analyzed during the current study.
